# Direct evidence of mitochondrial G-quadruplex DNA by using fluorescent anti-cancer agents

**DOI:** 10.1093/nar/gkv1061

**Published:** 2015-10-19

**Authors:** Wei-Chun Huang, Ting-Yuan Tseng, Ying-Ting Chen, Cheng-Chung Chang, Zi-Fu Wang, Chiung-Lin Wang, Tsu-Ning Hsu, Pei-Tzu Li, Chin-Tin Chen, Jing-Jer Lin, Pei-Jen Lou, Ta-Chau Chang

**Affiliations:** 1 Institute of Atomic and Molecular Sciences, Academia Sinica, Taipei 10617, Taiwan; 2 Institute of Biomedical Engineering, National Chung-Hsing University, Taichung 40227, Taiwan; 3 Department of Agricultural Chemistry, National Taiwan University, Taipei 10617, Taiwan; 4 Department of Biochemical Science and Technology, National Taiwan University, Taipei 10617, Taiwan; 5 Institute of Biochemistry and Molecular Biology, National Taiwan University College of Medicine, Taipei 10051, Taiwan; 6 Department of Otolaryngology, National Taiwan University Hospital and National Taiwan University College of Medicine, Taipei 10051, Taiwan

## Abstract

G-quadruplex (G4) is a promising target for anti-cancer treatment. In this paper, we provide the first evidence supporting the presence of G4 in the mitochondrial DNA (mtDNA) of live cells. The molecular engineering of a fluorescent G4 ligand, 3,6-bis(1-methyl-4-vinylpyridinium) carbazole diiodide (BMVC), can change its major cellular localization from the nucleus to the mitochondria in cancer cells, while remaining primarily in the cytoplasm of normal cells. A number of BMVC derivatives with sufficient mitochondrial uptake can induce cancer cell death without damaging normal cells. Fluorescence studies of these anti-cancer agents in live cells and in isolated mitochondria from HeLa cells have demonstrated that their major target is mtDNA. In this study, we use fluorescence lifetime imaging microscopy to verify the existence of mtDNA G4s in live cells. Bioactivity studies indicate that interactions between these anti-cancer agents and mtDNA G4 can suppress mitochondrial gene expression. This work underlines the importance of fluorescence in the monitoring of drug-target interactions in cells and illustrates the emerging development of drugs in which mtDNA G4 is the primary target.

## INTRODUCTION

A single-stranded G-rich sequence is capable of forming G-quadruplex (G4) via Hoogsteen hydrogen bonding under certain physiological conditions (1–3). G4s have been studied extensively due to their connection with human telomeres (4–8) and a number of promoter regions (9–13). Compared with G4s in nuclear DNA, little is known about G4s in mitochondrial DNA (mtDNA). Mitochondria are important not only in producing metabolic energy for live cells but also in regulating the process of cell death. Mitochondrial targeting has gained considerable attention for its potential to benefit the treatment of many diseases, including cancer. Human mtDNA contains 16569 bp, which encode 13 proteins essential to the respiratory chain as well as 2 rRNAs and 22 tRNA genes (14,15). Unlike mammalian nuclear DNA, mtDNA contains no introns or protective histones and presents a higher probability of forming G4s. A number of recent studies suggested that there are ∼200 putative G4 forming (PQF) sequences in mtDNA (16,17). However, to the best of our knowledge, no previous report has provided evidence to verify the existence of mtDNA G4 in cells. This is because nearly all of the G4 ligands have been used to interact with the G4s formed in human telomere or promoter regions, which are located in the nucleus of cells. Numerous mitochondrial-targeted agents have been developed for cancer therapy (18–22); however, no previous studies have reported on the use of G4 ligands to induce mitochondrial dysfunction via interaction with mtDNA G4 for cancer treatment. Thus, this study sought to verify the presence of mtDNA G4 in cells as well as to evaluate mtDNA G4 as a target in the development of drug for cancer therapy.

**Scheme 1.**
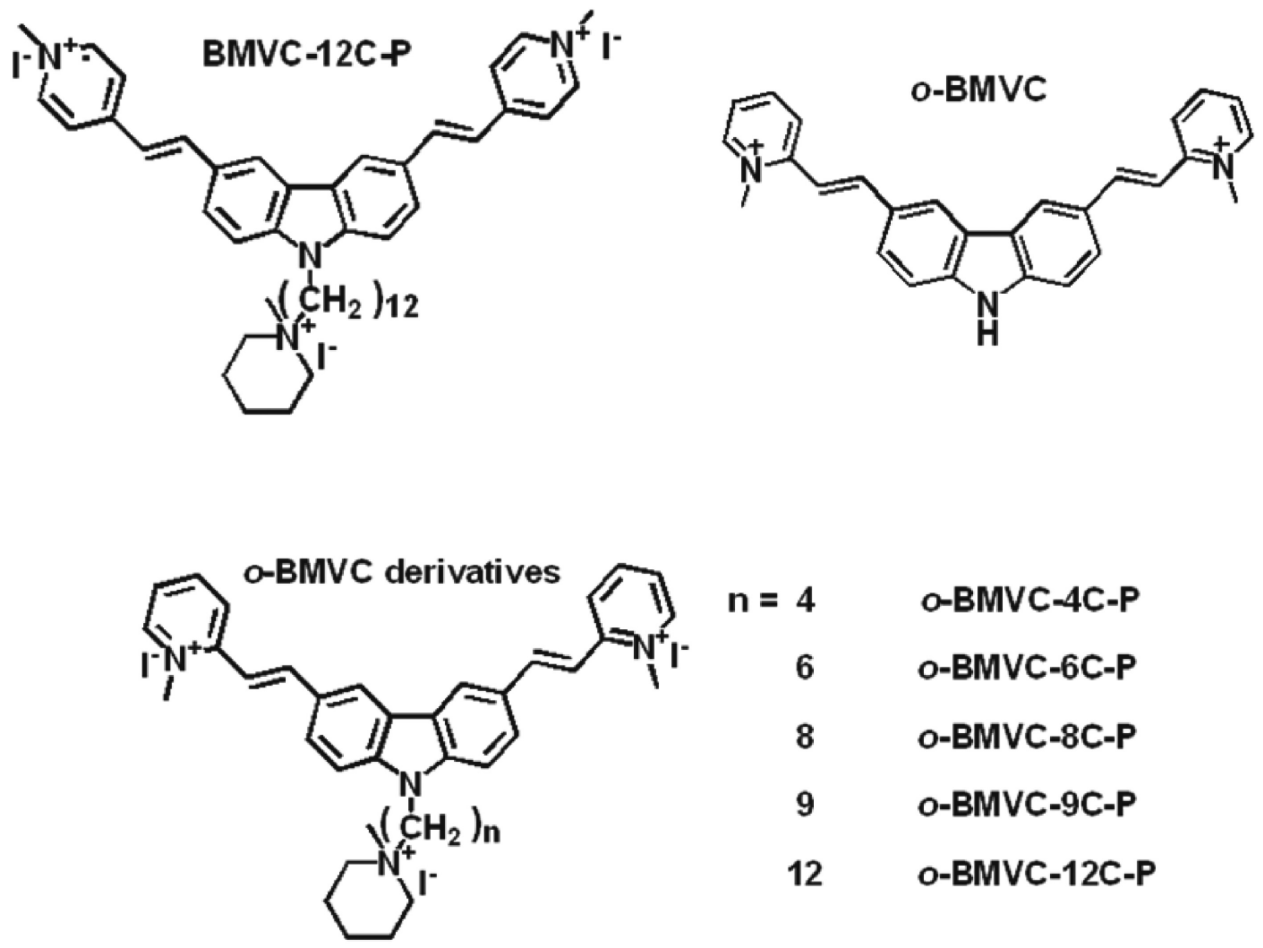
Chemical structures of BMVC-12C-P, *o*-BMVC, and *o*-BMVC derivatives.

Developing G4 ligand for the targeting of mtDNA requires that the G4 ligand be able to reach the cell mitochondria. The carbazole derivative 3,6-bis(1-methyl-4-vinylpyridinium iodide)-9-(1-(1-methyl-piperidinium iodide)dodecyl) carbazole (BMVC-12C-P) is a fluorescent anticancer agent. Fluorescent images have previously illustrated the accumulation of BMVC-12C-P primarily in the mitochondria of cancer cells. Furthermore, as little as 0.5 μM BMVC-12C-P can induce mitochondrial dysfunction resulting in cancer cell death with no risk of harming normal cells. However, another fluorescent G4 ligand, 3,6-bis(1-methyl-2-vinylpyridinium) carbazole diiodide (*o*-BMVC), which also accumulates in mitochondria (23), presents no appreciable cytotoxic effects on cancer cells. Nonetheless, the large contrast in decay times of *o*-BMVC fluorescence between the interaction with G4s and calf thymus DNA may be applied to the visualization of G4s located in cells (23). To examine the difference in cytotoxicity between BMVC-12C-P and *o*-BMVC, we synthesized a number of *o*-BMVC derivatives, including -4C-P, -6C-P, -8C-P, -9C-P and -12C-P. The chemical structures of BMVC-12C-P, *o*-BMVC and *o*-BMVC derivatives are presented in Scheme 1. The synthesis of these molecules is described in the Supplementary material.

Of interest is the fact that *o*-BMVC-nC-P molecules with n ≤ 6 show no appreciable effect on colony formation; however, *o*-BMVC-nC-P molecules with n ≥ 8 have been shown to inhibit colony formation. Fluorescence studies were conducted in conjunction with bioactivity assays of these molecules to elucidate their major targets and the underlying mechanism of these fluorescent anti-cancer agents. Moreover, our findings provide convincing evidence to support the existence of mtDNA G4s in live cells. Our findings also illustrate the ability of BMVC-12C-P and *o*-BMVC-12C-P to stabilize mtDNA G4s and thereby suppress mitochondrial gene expression. We believe that the development of G4 ligands capable of targeting mtDNA G4 is a unique approach to cancer research and perhaps other mitochondria related diseases as well (15,16,24).

## MATERIALS AND METHODS

### Fluorescence lifetime imaging microscopy (FLIM)

The setup of FLIM consists of a picosecond diode laser with emission wavelength of 470 nm with laser power of 5 mW (LDH470, PicoQuant, Germany) and a ∼70 ps pulse width for the excitation of *o-*BMVC under a scanning microscope (IX-71 and FV-300, Olympus, Japan) (23). The fluorescence of *o*-BMVC was collected using a 60X NA = 1.42 oil-immersion objective (PlanApoN, Olympus, Japan), passing through a 550/80 nm bandpass filter (Chorma, USA), and then detected using a fast PMT (PMA182, PicoQuant, Germany). The fluorescence lifetime was analyzed using a time-correlated single photon counting (TCSPC) module and software (TimeHarp-200 and SymPhoTime, PicoQuant, Germany). FLIM images were constructed from pixel-by-pixel lifetime information.

### Confocal microscopy

HeLa and MRC-5 cells were treated with 5 μM BMVC-12-P for 24 h, and co-stained with 20 nM Mitotracker Red CMXRos (Invitrogen, USA) for 20 min. Stained cells were washed twice using phosphate-buffered saline (PBS) and visualized using a confocal microscope (Leica TCS SP8).

### Xenograft mouse model

Approximately 2 × 10^6^ lung cancer H1299 cells were first injected s.c. into BALB/c mice (National Laboratory Animal Center, Taipei, Taiwan, Republic of China). Following the growth of tumors to ≈50–100 mm^3^, BMVC-12C-P was injected i.p. at a dose of 3 mg/kg every 3 d. The growth of the tumors was recorded using caliper measurements to determine the length (*L*) and width (*W*) of the s.c. tumor mass. The tumor volumes were estimated as *LW*^2^/2.

### Mitochondria isolation

A mitochondrial isolation kit (Thermo Scientific, USA) was used to isolate mitochondria from HeLa cells pretreated using BMVC derivatives. Briefly, pretreated cells were grown in the presence of 5 μM *o*-BMVC-12C-P or *o*-BMVC-6C-P for 24 h whereupon mitochondria was extracted from the cell suspension using their protocol recommended by the manufacturer (Thermo Scientific, USA). The extracted mitochondria were then filtered using a 5 μm column (Millipore, USA) to eliminate nucleus contaminations. The fluorescence of *o*-BMVC derivatives in isolated mitochondria was measured using a spectrofluorometer (LS-55, PerkinElmer, USA).

### Mitochondrial DNA depletion

To deplete mtDNA in A375 cells, long-term culture ≥70 passages were conducted in medium containing 50 ng/ml ethidium bromide (Sigma-Aldrich) as conventionally designated ρ^0^ cells (25). We used parental A375 cells and the mtDNA-depleted cells to determine whether the cytotoxicity is due to mtDNA targeted by *o*-BMVC-12C-P.

### RNA extraction and reverse transcription PCR

HeLa cells were treated with 1 μM BMVC-12C-P for 1 to 3 d and total RNA was extracted using Trizol reagent. The amount of total RNA from each sample was measured using a Nanophotometer (Implen GmbH, Germany) where 1 μg of RNA underwent reverse transcription into cDNA using SuperScript™III reverse transcriptase (Invitrogen, USA) with random hexamers primers. The resulting cDNA was mixed with 0.1 μM of each primer for final concentration, Taq 2X master mix (Invitrogen, USA) and ddH_2_O into 25 μl of volume. The reaction mixtures were incubated in a thermocycler under the following cycling conditions: denature 94°C for 5 min, followed by 30 cycles of 94°C for 45 s, 55°C for 30 s and 72°C for 45 s, and elongation 72°C for 10 min. PCR products were loaded onto 1.5% agarose gel in TBE buffer and stained using SYBR Gold (Invitrogen, USA).

### PCR stop assay

Polymerase stop assay was performed using a modified protocol previous reported (26). In short, the PCR stop assay was conducted by introducing different concentrations of compound (0–10 μM) into 25 μl solution containing 1X PCR buffer, 2 μM of each oligomer, 0.16 mM dNTP and 2.5 U Taq polymerase (Invitrogen, USA). Reaction mixtures were incubated in a thermocycler under the following cycling conditions: 94°C for 5 min followed by 30 cycles of 94°C for 30 s, 56°C for 30 s and 72°C for 30 s. Amplified products were resolved on 15% polyacrylamide gel and stained with SYBR Gold (Invitrogen, USA).

## RESULTS

### FLIM for visualizing G4s in live cells

The use of optical images in conjunction with fluorescent probes enabled the direct visualization of cellular uptake, localization, distribution and sometimes even probe-target interactions in living cells. We recently discovered that the G4 ligand *o*-BMVC has a longer fluorescence decay time when interacting with G4s (≥2.4 ns) than when interacting with calf thymus DNA (∼1.2 ns) *in vitro*. Furthermore, following the incubation of *o*-BMVC with CL1–0 cancer cells, we used time-discrete FLIM to detect a number of fluorescent spots with extended decay times (≥2.4 ns) in live cells (23). However, the possibility remains that this effect may be due to interactions with intracellular species other than G4 DNAs.

**Figure 1.**
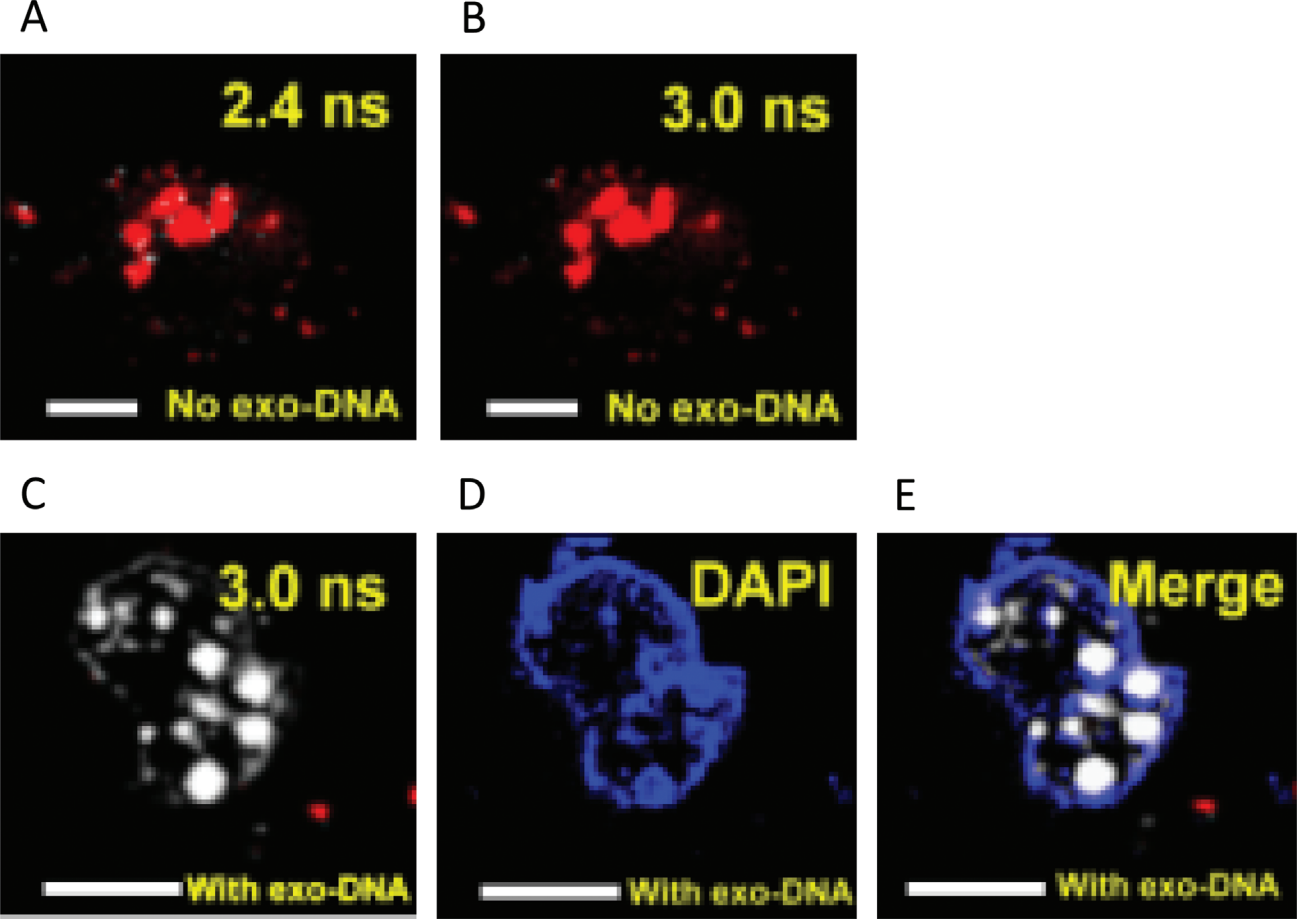
(**A**) FLIM (Fluorescence lifetime imaging microscopy) image of CL1–0 live cell incubated with 1 μM *o*-BMVC/lipofectamine complex. The results are presented in pseudocolors of white (decay time ≥ 2.4 ns) and red (decay time < 2.4 ns). (**B**) At the same time, the results are presented in pseudocolors of white (decay time ≥ 3.0 ns) and red (decay time < 3.0 ns). (**C**) A large number of fluorescent spots with extended decay time (≥3.0 ns) could be clearly observed in the nucleus of cancer cells, when lipofectamine was used to deliver *o*-BMVC and ssDNA of thrombin binding aptamer (TBA) simultaneously. (**D**) DAPI was used to stain the cell nuclei to confirm the delivery of TBA into the nucleus by lipofectamine. (**E**) The merged images. Scale bar is 10 μm.

In this work, we first demonstrated the effectiveness of FLIM as a tool for the visualization of G4 localization in live cells. The main target of *o*-BMVC is the mitochondria of CL1–0 cancer cells; therefore, we used lipofectamine for the delivery of *o*-BMVC into the nucleus (27). We applied a discrete time model to divide the image into two temporal regions, which made it possible to detect fluorescent spots with a longer decay time (≥2.4 ns) in the nucleus of live cells (Figure 1A). At the same time, fluorescent spots with an extended decay time (≥3.0 ns) were rarely observed in the nucleus of live cells (Figure 1B). It should be noted that a large number of fluorescent spots with extended decay time (≥3.0 ns) could be clearly observed in the nucleus of live cells, when lipofectamine was used to deliver *o*-BMVC and ssDNA of thrombin binding aptamer (TBA) (23) simultaneously (Figure 1C). In addition, DAPI was used to stain the cell nuclei to confirm the delivery of TBA into the nucleus by lipofectamine (Figure 1D). Figure 1E shows the overlay between Figure 1C and D. This finding unambiguously demonstrates that the bright fluorescent spots shown in Figure 1C can be attributed to the presence of exogenous TBA G4s in the nucleus of live cells. This conclusion is based on the fact that the fluorescence decay time of *o*-BMVC is ∼4.0 ns upon interacting with TBA G4s *in vitro* (23). Moreover, the structural conversion of TBA from ssDNA to G4 in the presence of potassium cations in the nucleus further supports the formation of endogenous G4 in live cells (Figure 1A).

### Fluorescent anti-cancer agents

**Figure 2.**
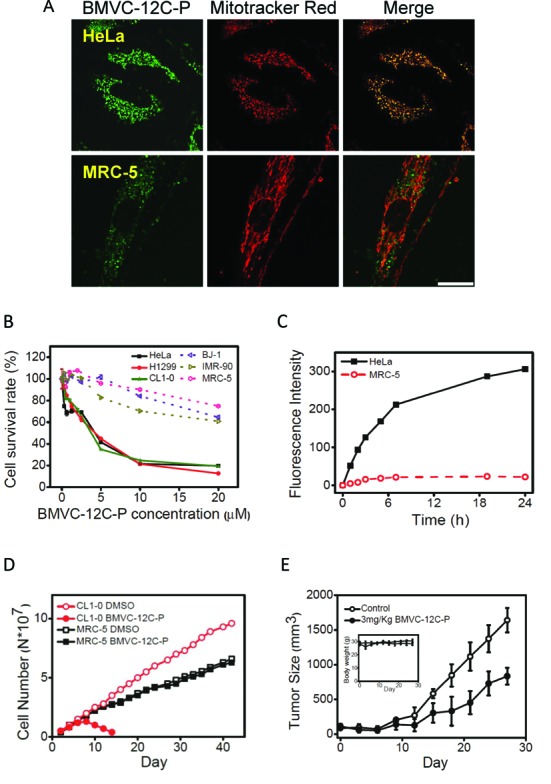
(**A**) Confocal images of HeLa and MRC-5 cells incubated with 5 μM BMVC-12C-P for 24 h (left) and then stained by 20 nM Mitotracker red (middle) together with their merges (right). The images were taken to visualize intracellular localization of BMVC-12C-P under optimized condition. Scale bar is 25 μm. (**B**) Cell viability assays of various cancer cell lines (solid line) and normal cell lines (dash line) after incubation with BMVC-12C-P from 0 μM to 20 μM for 72 h measured by MTT assay. (**C**) Time-dependent fluorescence intensity of 1 μM BMVC-12C-P treated HeLa and MRC-5 cells measured by flow cytometry. (**D**) Delayed anti-proliferation activity of BMVC-12C-P. CL1–0 cancer and MRC-5 normal cell lines were treated with 0.5 μM BMVC-12C-P. The numbers of cells were counted during the passages and the population doubling was determined. (**E**) BMVC-12C-P suppresses tumor growth. Nude mice (n = 4) were each injected with 1 × 10^6^ H1299 cells. BMVC-12C-P at 3 mg/kg was injected every 3 d. The tumor size and body weight (inset) were measured every 3 d.

*o*-BMVC is a G4 ligand with no appreciable cytotoxic effects on cancer cells. However, the BMVC derivative, BMVC-12C-P, is a fluorescent anti-cancer agent. The strong fluorescence of BMVC-12C-P accumulates primarily in the mitochondria of HeLa cancer cells, whereas weak fluorescence is distributed in the cytoplasm of MRC-5 normal fibroblasts (Figure 2A). Variations in the intensity of BMVC-12C-P fluorescence between cancer and normal cells were further confirmed using flow cytometry (Figure 2B). These variations can be attributed to a significant increase in the fluorescence of BMVC-12C-P upon interacting with DNA (Supplementary Figure S1) and also to a greater accumulation of BMVC-12C-P in the mitochondria of cancer cells than in normal cells.

Figure 2C illustrates the anti-cancer effects of BMVC-12C-P by showing MTT assays of HeLa, CL1–0, and H1299 cancer cells with MRC-5, IMR-90, and BJ1 normal fibroblasts following treatment with BMVC-12C-P over a period of 72 h. Again, flow cytometry was used to measure the mean fluorescence intensity of 1 μM BMVC-12C-P incubated with various cell lines for 24 h (Supplementary Figure S2). BMVC-12C-P accumulates mainly in the mitochondria of cancer cells, but is trapped mainly in the lysosome of normal cells (28). The large contrast in fluorescence intensity between cancer and normal cells can be explained by the interaction of BMVC-12C-P with mtDNA. This suggests that the difference in toxicity on cancer and normal cells lies in the greater uptake of the BMVC-12C-P in the mitochondria of cancer cells than in normal cells.

In addition, the cytotoxicity of 1 μM BMVC-12C-P on CL1–0, HeLa, and H1299 cancer cells was approximately 25%, with nearly negligible effects on normal MRC-5, IMR-90 and BJ1 fibroblasts (Figure 2C). Long-term treatment using 0.5 μM BMVC-12C-P halted the proliferation of CL1–0 after approximately three population doublings, whereas no appreciable effect was observed on normal MRC-5 fibroblasts (Figure 2D). This is a clear indication that BMVC-12C-P is capable of selectively inhibiting the proliferation of CL1–0 cancer cells without damaging MRC-5 normal fibroblasts. A tumor xenograft model was also used to examine the inhibition of tumor growth in nude mice following treatment with BMVC-12C-P (Figure 2E). Our results showed that the body weight of BMVC-12C-P treated mice was similar to that of the control mice, while the growth rate of tumors in BMVC-12C-P treated mice was significantly slower than that observed in the control mice. Taken together, these results suggest that BMVC-12C-P can inhibit the progression of tumor growth.

### Mechanism of cytotoxicity

**Figure 3.**
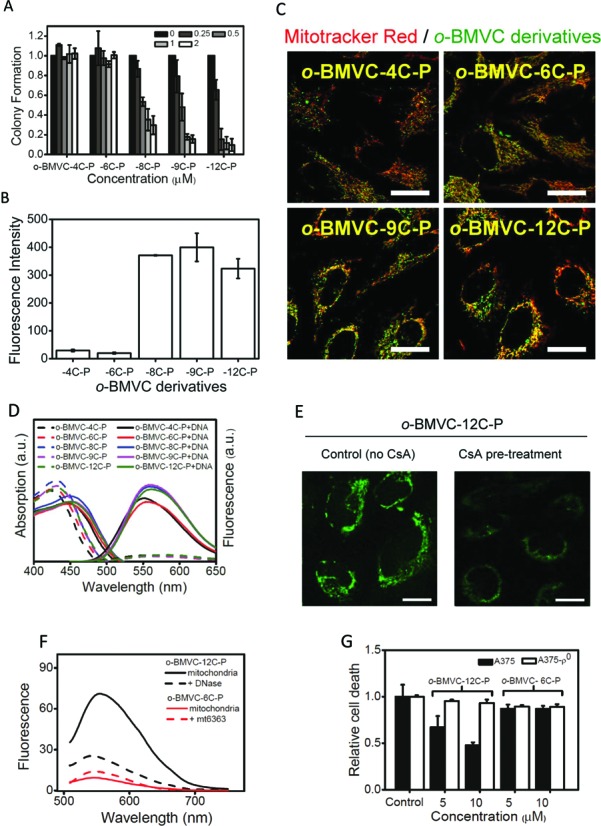
(**A**) Colony-forming ability of HeLa cells after *o*-BMVC derivatives treatment. Cells (1 × 10^3^) were seeded into each 6 cm culture plate for 24 h, then added different concentrations of each *o*-BMVC derivative to corresponding well for additional 5 d. Colonies were stained by crystal violet and quantified. (**B**) The mean fluorescence intensity of 1 μM *o*-BMVC derivatives incubated with HeLa cells for 24 h measured by flow cytometry. (**C**) To visualize the intracellular localizations of *o*-BMVC derivatives, confocal images of HeLa cells incubated with *o*-BMVC derivatives for 24 h and then co-stained with 20 nM Mitotracker Red for 20 min were taken under optimized conditions for each *o*-BMVC derivative. Red: Mitotracker Red; Green: *o*-BMVC derivatives; Yellow: merged. Scale bar is 25 μm. (**D**) Absorption and fluorescence spectra of *o*-BMVC derivatives and their complexes with calf thymus DNA. (**E**) Confocal images of HeLa cells without (left) and with (right) pre-treated 1 μM CsA for 24 h. After the medium were discarded, the cells were then incubated with 5 μM o-BMVC-12C-P for 4 h. Scale bar is 10 μm. (**F**) Fluorescence spectra of pre-treated *o*-BMVC-12C-P and *o*-BMVC-6C-P in the isolated mitochondria of HeLa cells. In addition, these isolated mitochondria were further treated with DNase and mt6363, respectively. (**G**) The major target is mtDNA supported by cell viability analyses of A375 wild-type and A375-ρ^0^ mtDNA-deficient cells. Both cells were treated with 5 and 10 μM of *o*-BMVC-12C-P and *o*-BMVC-6C-P for 72 h and then measured by MTT assay. A375-ρ^0^ cells were generated using ethidium bromide (EtBr) method to achieve mtDNA depletion.

In a previous study (28), structure-cellular localization analyses of BMVC-nC-P derivatives revealed that n ≥ 9 derivatives target to the mitochondria instead of the nucleus in cancer cells for n < 9 derivatives because longer C-chain results in higher lipophilicity. In this study, we synthesized a number of *o*-BMVC derivatives including -4C-P, -6C-P, -8C-P, -9C-P and -12C-P, to explain the difference in cytotoxicity between BMVC-12C-P and *o*-BMVC. We also hoped to preserve the large difference in the decay time of *o*-BMVC when interacting with G4s compared to its interaction with duplexes. BMVC-12C-P has a slightly longer fluorescence decay time when interacting with G4s (∼2.3 ns) than when interacting with calf thymus DNA (∼1.8 ns) *in vitro* (data not shown). Figure 3A illustrates the colony formation of HeLa cancer cells incubated with *o*-BMVC-4C-P, *o*-BMVC-6C-P, *o*-BMVC-8C-P, *o*-BMVC-9C-P and *o*-BMVC-12C-P. The *o*-BMVC-nC-P molecules with n ≤ 6 appear to have no appreciable effect on colony formation, while the *o*-BMVC-nC-P molecules with n ≥ 8 have a significant inhibitory effect on colony formation. Thus, the difference in cytotoxicity between *o*-BMVC-12C-P and *o*-BMVC-6C-P can be attributed to the length of the alkyl chains. We further found that the cytotoxic effect was directly proportional to the length of the alkyl chain for n ≥ 8. Supplementary Figure S3 presents the colony formation of CL1–0 and HeLa cancer cells with MRC-5 and BJ1 normal fibroblasts following incubation with BMVC-12C-P and *o*-BMVC-12C-P. These results demonstrate that *o*-BMVC-12C-P has stronger anticancer abilities than BMVC-12C-P.

Flow cytometry enables the quantitative measurement of fluorescence intensity in HeLa cells, which clearly illustrates a considerable difference in fluorescence intensity between the length of the alkyl chain with n ≤ 6 and n ≥ 8 in HeLa cells (Figure 3B). This finding was further confirmed by confocal images taken under the same experimental conditions (Supplementary Figure S4). In seeking to visualize intracellular localization of each *o*-BMVC derivative, confocal images taken under optimized conditions revealed the accumulation of these *o*-BMVC derivatives mainly in the mitochondria of the HeLa cells (Figure 3C).

To test whether this difference is due to differences in the fluorescence yield of these compounds, we measured the absorption and fluorescence spectra of *o*-BMVC derivatives and their complexes with calf thymus DNA in solution (Figure 3D). Supplementary Figure S5 presents the absorption coefficient and fluorescence quantum yield of each *o*-BMVC derivative and its interaction with LD12 duplex and two PQF mtDNA sequences. The fluorescence intensity of these *o*-BMVC derivatives increased 10–20 times upon interacting with calf thymus DNA. It appears that the ∼10-fold difference in fluorescence intensity between n ≤ 6 and n ≥ 8 *o*-BMVC derivatives in HeLa cells (Figure 3B) cannot be described by their fluorescence yields. It is far more likely to be the result in differences in uptake quantity, which suggests that an increase in mitochondrial uptake would lead to a subsequent increase in cytotoxicity.

The large difference in fluorescence intensity between *o*-BMVC-12C-P and *o*-BMVC-6C-P in HeLa cells suggests that mtDNA is the major target for the former molecule, but not for the latter. It is known that mtDNA is located in the mitochondrial matrix. Cyclosporine A (CsA) (29) is a permeability transition pore inhibitor, used to study whether *o*-BMVC-12C-P is able to reach the mitochondrial matrix. Indeed, the pre-treatment of CsA had been shown to block the penetration of *o*-BMVC-12C-P into the matrix, as indicated by far weaker fluorescence (Figure 3E). This finding is consistent with our hypothesis, i.e. mtDNA is the major target for *o*-BMVC-12C-P.

To verify this hypothesis, we isolated mitochondria from HeLa cells and measured the fluorescence intensity of *o*-BMVC-12C-P and *o*-BMVC-6C-P directly (Figure 3F). Our results clearly demonstrate that the intensity of fluorescence in *o*-BMVC-12C-P far exceeds the intensity in *o*-BMVC-6C-P due to differences in mitochondrial uptake. Furthermore, Figure 3F shows only a slight increase in the fluorescence of *o*-BMVC-6C-P following the addition of mt6363 into isolated mitochondria for a period of 24 h, which implies that the uptake of *o*-BMVC-6C-P into mitochondria is less pronounced. Figure 3F shows an appreciable decrease in the fluorescence of *o*-BMVC-12C-P following the addition of DNase to isolated mitochondria for a period of 24 h. This decrease in the intensity of fluorescence can be explained by the fact that DNase is able to degrade mtDNA, which provides convincing evidence to support our hypothesis that mtDNA is the major target of *o*-BMVC-12C-P.

Considering that the interaction of *o*-BMVC-12C-P with mtDNA is able to inhibit the proliferation of cancer cells, one would expect that the cytotoxic effects of *o*-BMVC-12C-P should be less potent in mtDNA-deficient cells than in corresponding wild-type cells (30). We thus employed wild-type A375 cancer cells and mtDNA-deficient A375 cells (A375-ρ^0^) to verify the role of mtDNA in *o*-BMVC-12C-P-induced cell death. As shown in Figure 3G, the cytotoxic effects observed in wild-type A375 cells was greatly reduced in A375-ρ^0^ cells following treatment with *o*-BMVC-12C-P, whereas no appreciable cytotoxic effect was observed in wild-type A375 or A375-ρ^0^ cells following *o*-BMVC-6C-P treatment. The evidence provided by fluorescence images and bioactive assays clearly indicates that mtDNA is the major target of *o*-BMVC-12C-P but not of *o*-BMVC-6C-P. These results further suggest that the alkyl chain length in *o*-BMVC derivatives plays a critical role in inducing mitochondrial dysfunction.

### Formation of mtDNA G4s stabilized by G4 ligands *in vitro*

Dong *et al*. (16) identified nine PQF sequences with three layers of G-quartet (3-G) and 178 PQF sequences with two layers of G-quartet (2-G). Among these, each motif is less than 33 nt long. We first used circular dichroism (CD) spectra to characterize the formation of G4s from 10 PQF sequences of mtDNA (Table 1 and Supplementary Figure S6). Among them, only mt15653 is unable to form G4, as revealed by the absence of ∼265 nm or ∼290 nm CD bands, while the others are capable of forming G4s in 150 mM K^+^ solution. This difference is probably due to a long loop with 16 nt in mt15653 with 2-G. Despite the fact that mt10252 also contains a long loop with 16 nt, it is able to form G4 with 3-G because this configuration is generally far more stable than G4 with 2-G. Our results suggest that most PQF sequences of mtDNA are able to form G4s under physiological conditions *in vitro*.

**Table 1.** Sequences of mitochondria DNA studied in CD spectra and FLIM

Sequence (5′→3′)	Abbreviations	Location
5′-G_12_TTTGATGTG_3_TTG_3_	mt377	16166–16193
5′-G_3_AGATAGTTG_2_TATTAG_2_ATTAG_2_	mt714	15831–15856
5′-G_3_CTTGATGTG_4_AG_4_TGTTTAAG_3_	mt1015	15516–15545
5′-AG_3_ACGCG_3_CG_5_ATATAG_3_T	mt6363	10182–10207
5′-G_3_AG_2_TAG_2_TG_2_	mt8095	8463–8475
5′-G_2_CGTAG_2_TTTG_2_TCTAG_3_	mt9438	7113–7132
5′-G_3_TG_3_AgTAGTTCCCTGCTAAG_3_AG_3_	mt10252	6289–6318
5′-G_3_CCAG_4_ATTAATTAGTACG_3_AAG_3_	mt12086	4455–4484
5′-G_3_TTAATCGTGTGACCGCG_2_TG_2_CTG_2_	mt15653	890–917
5′-GAAGCG_5_AG_7_TTTG_2_TG_2_AAAT	mt16250	291–320

**Figure 4.**
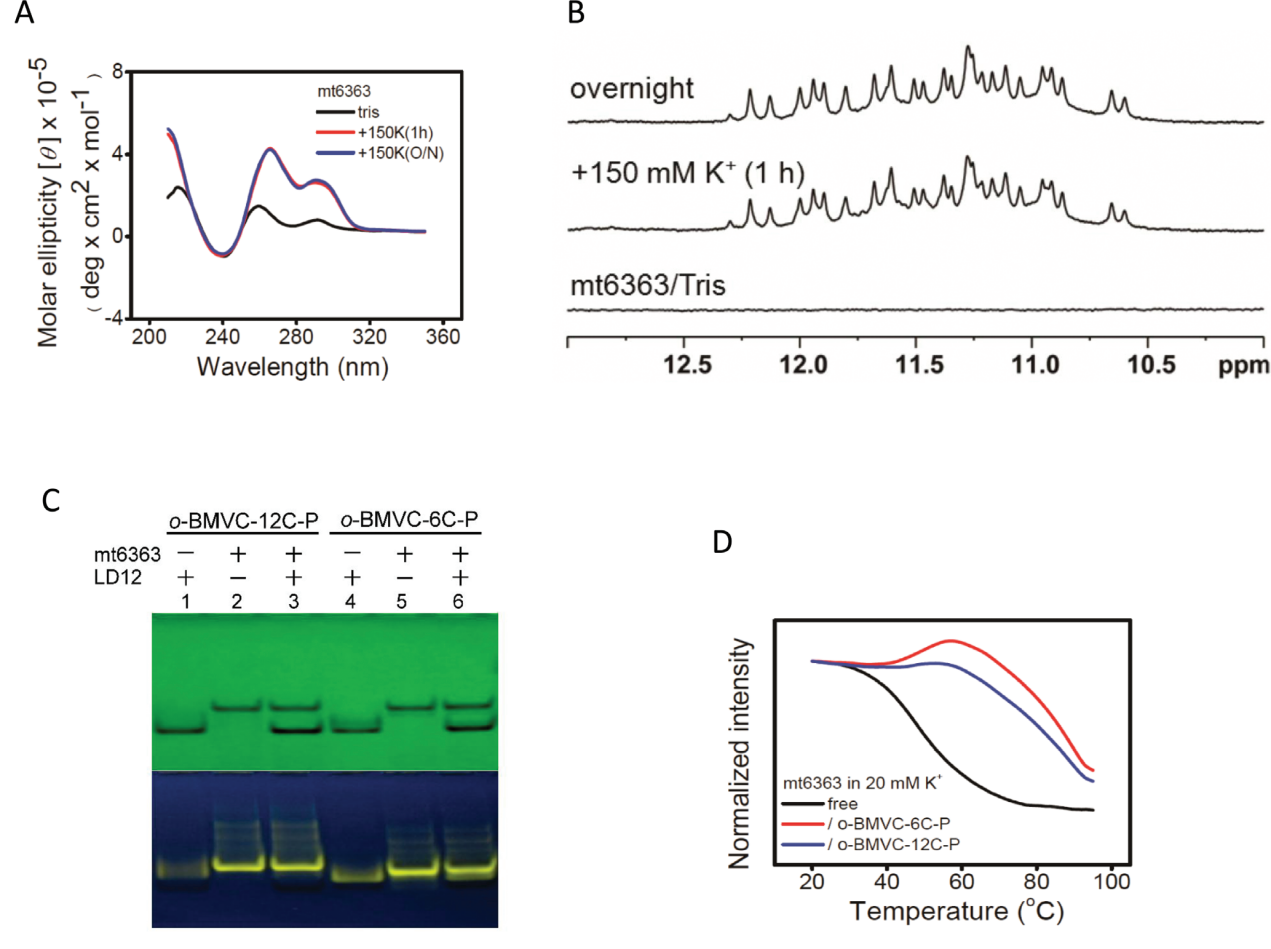
(**A**) CD spectra of 4 μM mt6363 and (**B**) NMR spectra of 100 μM mt6363 annealed in 10 mM Tris buffer, after the addition of 150 mM K^+^ for 1 h, and overnight. (**C**) UV shadowing of gel assays of (i) LD12 + *o*-BMVC-12C-P, (ii) mt6363 + *o*-BMVC-12C-P, (iii) LD12 + mt6363 + *o*-BMVC-12C-P, (iv) LD12 + *o*-BMVC-6C-P, (v) mt6363 + *o*-BMVC-6C-P, (vi) LD12 + mt6363 + *o*-BMVC-6C-P (750 pmol per well). (**D**) The melting curves monitored at 265 nm of 4 μM mt6363 and their complexes with 8 μM *o*-BMVC-6C-P and *o*-BMVC-12C-P in 20 mM K^+^ solution.

CD spectra (Figure 4A) and imino proton NMR spectra (Figure 4B) illustrate the G4 formation of the mt6363 sequence in the presence of K^+^. The aromatic and methyl NMR spectra can also be found in Supplementary Figure S7. The mt6363 sequence is located at positions 10182–10207 in the reversed mtDNA of the ND3 gene. Specifically, there are 24 imino proton NMR signals of mt6363 in the region of 10–12.5 ppm, indicating that two different G4s co-exist with 3-G *in vitro*. Competition gel analysis (23) was also conducted to determine whether *o*-BMVC-12C-P and *o*-BMVC-6C-P preferentially bind with mt6363 G4 or the LD12 duplex (Figure 4C). The relative quantities of duplex remaining in the mixture and the considerable weakening of pre-stained duplex suggest that these G4 ligands have a higher binding preference for mt6363 G4 than for the LD12 duplex. We further investigated the ability of these ligands to stabilize the G4s of mt6363. Figure 4D presents the CD melting curves of mt6363 at 265 nm in the absence and presence of *o*-BMVC-12C-P and *o*-BMVC-6C-P. The melting temperature (Tm) of G4 increased by ∼30°C upon interacting with these compounds, indicating that these G4 ligands are indeed able to stabilize the G4s formed by mt6363. It appears that these *o*-BMVC derivatives are all good G4 ligands, but not necessarily anti-cancer agents.

Supplementary Figure S8 illustrates the similarities in the results of CD, NMR, gel analysis and Tm on mt9438 with 2-G. The mt9438 is located at positions 7113–7132 in the reversed mtDNA of the COX I gene. The two positive CD bands at ∼245 and ∼290 nm together with a negative CD band at ∼265 nm and the imino proton NMR signals indicate that mt9438 is capable of forming an anti-parallel G4. Of interest is the fact that the Tm of mt9438 is ∼35°C in 20 mM K^+^ solution, which is slightly below body temperature; therefore, the G4 equilibrium is inclined toward an unfolded state. It is important to note that the addition of these G4 ligands can greatly stabilize and increase the folded G4 of mt9438. Moreover, the fluorescence titration of *o*-BMVC-6C-P and *o*-BMVC-12C-P upon interacting with mt9438 G4 and LD12 duplex DNA shows that the binding preference of these ligands to the G4 is approximately one order of magnitude stronger than to the duplex (Supplementary Figure S9). The formation of G4s with 2-G deserves more attention due to the relatively low Tm of G4s with 2-G in near physiological conditions.

### Existence of G4s in mitochondria

**Figure 5.**
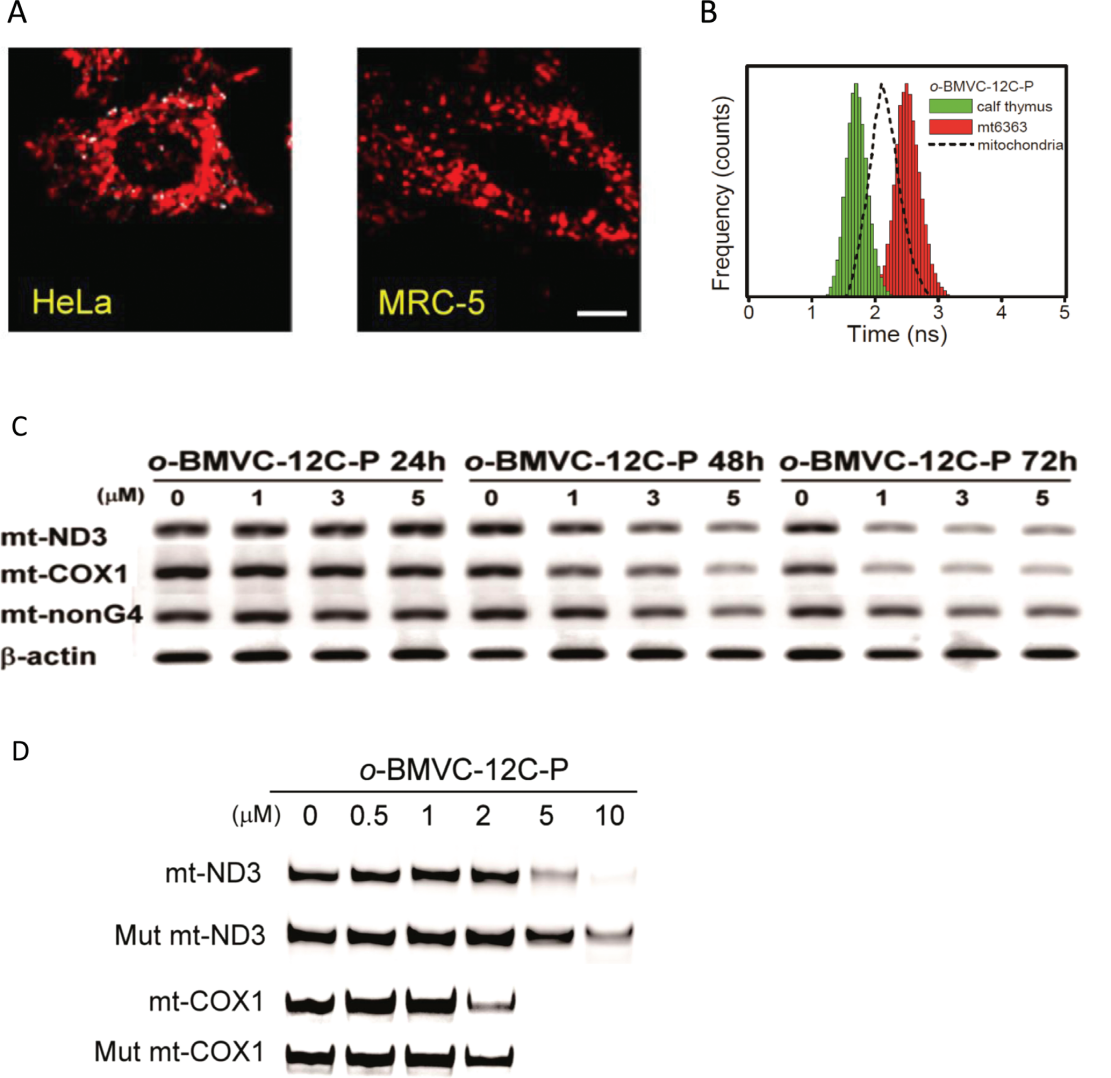
(**A**) FLIM images of live cells incubated with 1 μM *o*-BMVC-12C-P overnight. For clarity, the images were presented in pseudocolors of white (decay time ≥ 2.4 ns) resulting from interaction with G4s and red (decay time < 2.4 ns) due to interaction with others. Several white spots were detected in the time-discrete image of HeLa cancer cells, but not observed in the time-discrete image of MRC-5 normal cells. Scale bar is 10 μm. (**B**) The histograms of the fluorescence decay time of 0.2 μM *o*-BMVC-12C-P upon interaction with calf thymus (green) and mt6363 (red) together with *o*-BMVC-12C-P in isolated mitochondria (black). (**C**) Reverse transcription RT-PCR was used to evaluate the suppression of ND3 and COX I expression by *o*-BMVC-12C-P. HeLa cells were incubated with *o*-BMVC-12C-P at different concentrations for 1–3 d. Here β-actin and a non-G4 forming gene sequence were used as controls. (**D**) PCR stop assay was used to determine whether the suppression of ND3 and COX I expression is due to the stabilization of the G4s formed by mtDNA of ND3 and COX I by *o*-BMVC-12C-P. The paused bands indicated the G4 stabilization by the ligand. The mutated sequences were designed specifically for not able to form G4.

Figure 5A presents a time-discrete FLIM image of *o*-BMVC-12C-P to reveal the possible existence of G4s in HeLa cancer cells and MRC-5 normal cells under the same experimental conditions. Several white spots characterized by fluorescence decay time (≥2.4 ns) were detected in the study of HeLa cancer cells (24/24), but they were not observed in MRC-5 normal cells (15/15). The absence of white spots in the normal cells can be explained by relatively far lower uptake of *o*-BMVC-12C-P in the mitochondria of MRC-5 normal cells. This is because the *o*-BMVC-12C-P localizes primarily in the lysosome of normal cells. Nevertheless, the detection of the white spots in cancer cells supports the existence of G4s in cancer cells, which is very likely contributed by mtDNA G4s.

Figure 5B presents a histogram of the decay time of *o*-BMVC-12C-P fluorescence upon interacting with calf thymus DNA and mt6363 as well as in isolated mitochondria of HeLa cells. These findings confirm the existence of mtDNA G4s. The decay time of *o*-BMVC-12C-P was ∼1.7 ns upon interacting with calf thymus DNA and ∼2.5 ns in the presence of mt6363 G4. As shown in Supplementary Figure S10, similar results were obtained for fluorescence decay time in mt9438 (∼3.0 ns), mt1015 (∼3.3 ns), Tel23 (∼2.7 ns) and LD12 (∼1.7 ns). Despite the fact that the binding preference of *o*-BMVC-12C-P to the G4 is approximately one order of magnitude higher than it is to the duplex (Supplementary Figure S9), it is not surprising to detect a contribution from the duplex due to the relatively large quantities of duplex over G4 structures in mtDNA. Of importance is the detection of the extended decay time (≥2.4 ns) in isolated mitochondria. DNase analysis indicates that the fluorescence intensity is mainly due to the binding of *o*-BMVC-12C-P to mtDNA (Figure 3F). Accordingly, detecting the contribution from the interaction with mtDNA characterized by longer decay time (≥2.4 ns) strongly supports the existence of G4s in the mtDNA of cells.

We further conducted RT-PCR to screen the most prominent effects resulting from the gene expression of ND2, ND3, ND5, ND6, COX I, COX II, ATP8 and CYT B, following treatment with *o*-BMVC-12C-P (Supplementary Table S1). Figure 5C clearly illustrates the reduction in COX I and ND3 gene expression following treatment with *o*-BMVC-12C-P as well as other minor effects on non-G4 mtDNA expression. These findings suggest that *o*-BMVC-12C-P could suppress the expression of ND3 and COX I via the targeting of G4s in mtDNA. In addition, we employed a different G4 ligand, TMPyP4, to test the gene expression of ND3 and COX1 using RT-PCR (Supplementary Figure S11). TMPyP4 is a well-known G4 ligand that interacts with c-MYC promoter oncogene for gene suppression (31). Our finding indicates that TMPyP4 shows no appreciable effect on either form of gene expressions, due to the fact that TMPyP4 targets mainly the nucleus of cells.

Figure 5D presents the results of PCR stop assays using mt6363 and mt9438 G4s treated with *o*-BMVC-12C-P as well as their mutants containing non-G4 forming sequences. Our aim was to determine whether the suppression of ND3 and COX I expression is due to the stabilization of the G4s of mt6363 (ND3) and mt9438 (COX I) by *o*-BMVC-12C-P. Our results provide substantive evidence of an interaction between *o*-BMVC-12C-P and mtDNA G4s. The combined results of fluorescence studies and bioactive assays provide strong evidence supporting the existence of G4s in mitochondria. Furthermore, these data suggest that the mtDNA G4s stabilized by *o*-BMVC-12C-P play an important role in suppressing ND3 and COX I expression.

## DISCUSSION

BMVC-12C-P, *o*-BMVC-12C-P, *o*-BMVC-6C-P and *o*-BMVC play dual roles as fluorescent probes and G4 stabilizers *in vitro*. BMVC-12C-P and *o*-BMVC-12C-P have been shown to induce cancer cell death; however, *o*-BMVC-6C-P and *o*-BMVC have not. The former two are fluorescent anti-cancer agents, while the latter two are fluorescent G4 ligands. Our investigation using fluorescence revealed the mechanism that leads to the differences in cytotoxicity and the major target in mitochondria. Briefly, *o*-BMVC-6C-P presents the same G4 stabilization characteristics as *o*-BMVC-12C-P does and also targets mitochondria; however, it presents weak fluorescence and has no cytotoxic effects on cancer cells. This implies that the effects of the interactions between *o*-BMVC-6C-P and other biomolecules are limited. Our fluorescence study of isolated mitochondria suggests that mitochondrial uptake (associated with lipophilicity (28) and regulated by the length of alkyl chain) is the mechanism primarily responsible for differences in cytotoxicity between these G4 ligands. Specifically, the strong fluorescence of *o*-BMVC-12C-P in mitochondria is due to (1) the interaction with mtDNA and (2) the fact that a sufficient quantity of *o*-BMVC-12C-P can suppress mtDNA gene expression and eventually induce cell death. This study demonstrated the importance of fluorescent anti-cancer agents in therapeutic applications and also helped to elucidate the underlying mechanisms that could be exploited through the development of effective anti-tumor agents.

Obtaining proof for the existence of G4 in living cells has been a long-term challenge (32); however, recent studies on the visualization of exogenous and endogenous G4s in human cells firmly support the existence of G4s in live cells (8,23,33–35) and fixed cells (36–38). In addition, the formation of G4s has been implicated in many biological processes, which has led to the suggestion that the topology of G4 may provide a novel therapeutic target. Our results from fluorescence studies and bioactivity assays provide convincing evidence to support the existence of G4 in isolated mitochondria as well as in the mitochondria of live cells.

The fact that there are ∼200 PQF sequences of mtDNA (16,17) underlines the importance of identifying all G4s that may be involved in G4 ligand-induced cell death and elucidating how the G4 ligand affects mtDNA function. In this study, BMVC-12C-P and *o*-BMVC-12C-P were shown to stabilize the mtDNA G4s; however, the results of RT-PCR provide evidence of mtDNA gene-dependent suppression with more pronounced suppression of ND3 and COXI. PCR stop assays suggest that G4s formed by mt6363 (in ND3) and mt9438 (in COX I) are at least partially the targets of BMVC-12C-P and *o*-BMVC-12C-P. This makes it possible to use RT-PCR in conjunction with PCR stop assays for the identification of mtDNA G4s involved in G4 ligand induced cell death.

Previous reports have shown a high degree of correlation between the PQF sequences of mtDNA and mtDNA deletion, which could disrupt genomic stability in mitochondria (16,17). It has also been reported that a DNA/RNA hybrid G4 of a conserved sequence block II (CSB II) may play a regulatory role in transcriptional termination (38–41). Thus, these studies indicate the existence of G4s and the potential roles played by mtDNA G4s in mitochondrial function. The fact that numerous mitochondrial diseases are related to G4 DNA-forming sequences means that a rational drug design capable of manipulating or even disrupting G4 structures could be an important avenue for the further development of treatment modalities. We believe that mtDNA G4 is a promising target for mitochondrial medicine.

In summary, our findings illustrate the importance of fluorescence as a tool for exposing targets in live cells, detecting G4 in mitochondria, unraveling the anti-cancer mechanisms of agent in cells and guiding drug development. Specifically, this work provides very strong evidence to support the existence of G4s in the mitochondria of live cells and presents the first example of a G4 anti-cancer agent that targets the mtDNA of cancer cells and eventually causes cell death.

## Supplementary Material

SUPPLEMENTARY DATA
