# Supplementary material for: Direct evidence of mitochondrial G-quadruplex DNA by using fluorescent anti-cancer agents
Source: Nucleic Acids Res. 2015 Oct 19;43(21):10102–13. doi: 10.1093/nar/gkv1061 (PMC4666356; doi:10.1093/nar/gkv1061)
Supplement: SUPPLEMENTARY DATA [file supp_43_21_10102__index.html]

Direct evidence of mitochondrial G-quadruplex DNA by using fluorescent anti-cancer agents — SUPPLEMENTARY DATA 

# Direct evidence of mitochondrial G-quadruplex DNA by using fluorescent anti-cancer agents

## SUPPLEMENTARY DATA

- SUPPLEMENTARY DATA
