# Supplementary material for: Direct evidence of mitochondrial G-quadruplex DNA by using fluorescent anti-cancer agents
Source: Nucleic Acids Res. 2015 Oct 19;43(21):10102–13. doi: 10.1093/nar/gkv1061 (PMC4666356; doi:10.1093/nar/gkv1061)
Supplement: SUPPLEMENTARY DATA [file supp_gkv1061_nar-01824-f-2015-File002.pdf]

Supplementary

Direct evidence of mitochondrial G-quadruplex DNA by using fluorescent anti-cancer agents

Wei-Chun Huang,[1] Ting-Yuan Tseng,[1] Ying-Ting Chen,[1] Cheng-Chung Chang,[2] Zi-Fu Wang,[1] Chiung-Lin Wang,[1] Tsu-Ning Hsu,[1,3] Pei-Tzu Li,[4] Chin-Tin Chen,[4] Jing-Jer Lin,[5] Pei-Jen Lou,[6] Ta-Chau Chang,[1,*]

[1] Institute of Atomic and Molecular Sciences, Academia Sinica, Taipei, Taiwan

[2] Institute of Biomedical Engineering, National Chung-Hsing University, Taichung, Taiwan

[3] Department of Agricultural Chemistry, National Taiwan University, Taipei, Taiwan

[4] Department of Biochemical Science and Technology, National Taiwan University, Taipei, Taiwan

[5] Institute of Biochemistry and Molecular Biology, National Taiwan University College of Medicine, Taipei, Taiwan

[6] Department of Otolaryngology, National Taiwan University Hospital and National Taiwan University College of Medicine, Taipei, Taiwan

* To whom correspondence should be addressed.

E-mail: tcchang@po.iams.sinica.edu.tw

Methods and Materials

Chemicals

Synthesis of compounds: The syntheses of 9-substituted ***o*-BMVC** derivatives were shown in Scheme S1. All Compounds (**2-4C-P, 2-6C-P, 2-8C-P, 2-9C-P, 2-12C-P**) were synthesized from 3,6-dibromocarbazole (**1,** 2 g, 6.15 mM, Aldrich) through 9-position substituting by sodium hydride (0.295 g, 12.3 mM, Aldrich) in DMF (20 mL) under nitrogen gas condition. Dibromoalkanes (Br-R-Br, 100 mM) were then added and the mixtures were refluxed for 12 h. After cooling and quenching the excess sodium hydride with methanol, the solution was extracted with H_2O/ethyl acetate twice and the organic layer dried by $MgSO_4$. The products (**2-4C-P, 2-6C-P, 2-8C-P, 2-9C-P, 2-12C-P**) were collected by flash column (silica, hexane/ethyl acetate. 2/1, v/v). Piperidine attaches to compound **3** was conveniently obtained by way of refluxing **2** (5.0 mM) and piperidine (0.5 mL, Aldrich) in ethanol (20 mL) for 6 h with trace of sodium iodide. The solvent was evaporated in vacuum and the residue purified via column chromatography (silica, hexane/ethyl acetate. 1/2, v/v) to collect the yellow products (**3-4C-P, 3-6C-P, 3-8C-P, 3-9C-P, 3-12C-P**). Then, the reactants **3** coupled with 2-vinylpyridine at mixed powders of Palladium (II) acetate / tri-*o*-tolylphosphine under the triethylamine / acetonitrile solvent pairs in high-pressure system. The bottle was sealed after bubbling 10 min with nitrogen. After keeping the system under ~105 ^{o}C for two days, the precipitant was collected and then extracted with H_2O/CH_2Cl_2 twice. The CH_2Cl_2 layer were dried by $MgSO_4$ and then filtered. Crude products were purified by flash column chromatography with acetone/n-hexane as eluent gradient to collect the compounds **4-4C-P, 4-6C-P, 4-8C-P, 4-9C-P** and **4-12C-P**. The orange-red powders (9-substituted ***o*-BMVC** derivatives, ***o*-BMVC-4C-P, *o*-BMVC-6C-P, *o*-BMVC-8C-P, *o*-BMVC-9C-P** and ***o*-BMVC-12C-P**) were collected in very good yield after refluxing the compound **4** with excess CH_3I in DMF.

3,6-Dibromo-9-(1-bromobutyl) carbazole (2-4C-P) (Yield: 58%, Mw: 462.02), ^{1}H NMR (400 MHz, $CDCl_3$): 8.07 (d, *J* = 1.8Hz, 2H), 7.55 (dd, *J* = 1.8, 8.4Hz, 2H), 7.21 (d, *J* = 8.4Hz, 2H), 4.20 (t, *J* = 6.2Hz, 2H), 3.35 (t, *J* = 6.6Hz, 2H), 1.95 (m, 2H), 1.84 (m, 2H).

3,6-Dibromo-9-(1-(piperidin-1-yl)butyl) carbazole (3-4C-P) (Yield: 92%, Mw: 464.03), ^{1}H NMR (400 MHz, $CDCl_3$): 7.86 (d, *J* = 1.89Hz, 2H), 7.35 (dd, *J* = 1.8,

8.7Hz, 2H), 7.03 (d, *J* = 8.7Hz, 2H), 3.95 (t, *J* = 7.2Hz, 2H), 2.15 (m, 4H), 2.10 (t, *J* = 7.5Hz, 2H), 1.63 (t, *J* = 7.2Hz, 2H), 1.44 (m, 4H), 1.36 (m, 2H), 1.32 (m, 2H).

3,6-Bis-(2-vinylpyridine)-9-(1-(piperidin-1-yl)butyl) carbazole (4-4C-P) (Yield: 71%, Mw: 512.69), ^{1}H NMR (400 MHz, $CDCl_3$): 8.59 (d, *J* = 4.5Hz, 4H), 8.29 (d, *J* = 1.2Hz, 2H), 7.82 (d, *J* =16 Hz, 2H), 7.70 (dd, *J* = 2.0, 8.7Hz, 2H), 7.52 (dd, *J* = 2.0, 7.6 Hz, 2H), 7.43 (d, *J* = 9.0Hz, 2H), 7.40 (d, *J* = 4.8Hz, 2H), 7.26 (d, *J* = 16.2Hz, 2H), 7.06 (d, *J* = 7.2 Hz, 2H), 4.35 (t, *J* = 7.2Hz, 2H), 2.32 (m, 8H), 1.93 (t, *J* = 7.5Hz, 2H), 1.56 (m, 4H), 1.44 (m, 2H). EA ($C_{35}H_{36}N_4$): calc. (obs %) C: 81.99 (81.84), H: 7.08 (7.06), N: 10.93 (10.90)

3,6-Bis-(1-methyl-2-vinylpyridium iodide)-9-(1-(1-methyl-piperidinium iodide)butyl) carbazole (*o*-BMVC-4C-P): (Yield: 82%, Mw: 994.61), ^{1}H NMR (400 MHz, DMSO-d6) δ: 8.98 (s, 2H), 8.89 (d, *J* = 6.4Hz, 2H), 8.56 (d, *J* = 8.4Hz, 2H), 8.46 (t, *J* = 8Hz, 2H), 8.16 (d, *J* = 16Hz, 2H), 8.03 (d, *J* = 8.8Hz, 2H), 7.87 (t, 2H), 7.86 (d, *J* = 8.4Hz, 2H), 7.67 (d, *J* = 16Hz, 2H), 4.55 (t, 2H), 4.45 (s, 6H), 3.37 (m, 6H), 2.99 (s, 3H), 1.85 (m, 4H), 1.77 (m, 4H), 1.52 (m, 2H). ^{13}C NMR (500 MHz, DMSO-d6): 144.65, 144.37, 142.67, 128.36, 127.41, 124.85, 124.81, 123.23, 122.31, 115.11, 111.09, 60.46, 46.97, 26.29, 21.38, 21.13, 19.76.

EA ($C_{38}H_{45}I_3N_4•H_2O$): calc. (obs %) C: 48.17 (48.09), H: 4.89 (4.86), N: 5.91 (5.88).

3,6-Dibromo-9-(1-bromohexyl) carbazole (2-6C-P) (Yield: 72%, Mw: 490.02), ^{1}H NMR (400 MHz, $CDCl_3$) δ: 8.08 (d, *J* = 2.0Hz, 2H), 7.52 (dd, *J* = 2.0, 8.8 Hz, 2H), 7.21 (d, *J* = 8.4Hz, 2H), 4.21 (t, *J* = 7.2Hz, 2H), 3.35 (t, *J* = 7.2Hz, 2H), 1.81 (m, 4H), 1.35 (m, 2H) , 1.27 (m, 2H).

3,6-Dibromo-9-(1-(piperidin-1-yl)hexyl) carbazole (3-6C-P) (Yield: 76%, Mw: 492.03), ^{1}H NMR (400 MHz, $CDCl_3$) δ: 8.11 (d, *J* = 2.0Hz, 2H), 7.53 (dd, *J* = 2.0, 8.4Hz, 2H), 7.25 (d, *J* = 8.4Hz, 2H), 4.21 (t, *J* = 7.6Hz, 2H), 2.34 (m, 4H), 2.24 (t, *J* = 8.4Hz, 2H), 1.80 (t, *J* = 8.4Hz, 2H), 1.61 (m, 4H), 1.50 (m, 4H), 1.39 (m, 2H), 1.25 (m, 2H).

3,6-Bis-(2-vinylpyridine)-9-(1-(piperidin-1-yl)hexyl) carbazole (4-6C-P) (Yield: 68%, Mw: 560.69), ^{1}H NMR (400 MHz, $CDCl_3$) δ: 8.60 (d, *J* =4.8 Hz, 2H), 8.29 (d, *J* =2 Hz, 2H), 7.82 (d, *J* =16 Hz, 2H), 7.73 (dd, *J* = 2.0, 8.8 Hz, 2H), 7.65 (dd, *J* = 2.0, 7.6 Hz, 2H), 7.43 (d, *J* = 8 Hz, 2H), 7.36 (d, *J* = 8.8 Hz, 2H), 7.24 (d, *J* = 16 Hz, 2H), 7.12 (d, *J* = 7.2 Hz, 2H), 4.25 (t, *J* = 7.2 Hz, 2H), 2.35 (m, 4H), 2.25(t, *J* = 7.6 Hz, 2H), 1.87 (t, *J* = 7.2 Hz, 2H), 1.57 (m, 2H), 1.51 (m, 2H) , 1.40 (m, 2H) , 1.31(m, 2H).

3,6-Bis-(1-methyl-2-vinylpyridium iodide)-9-(1-(1-methyl-piperidinium iodide)hexyl) carbazole (*o*-BMVC-6C-P) (Yield: 90%, Mw: 966.56), ^{1}H NMR (400 MHz, DMSO-d6) δ: 8.96 (s, 2H), 8.89 (d, *J* = 8.4Hz, 2H), 8.58 (d, *J* = 8.4Hz, 2H), 8.49 (t, *J* = 7.6 Hz, 2H), 8.18 (d, *J* = 16Hz, 2H), 8.06 (d, *J* = 8.8Hz, 2H), 7.87 (t, *J* = 6.8Hz, 2H), 7.83 (d, *J* = 8.8Hz, 2H), 7.65 (d, *J* = 16Hz, 2H), 4.53 (t, *J* = 6.8 Hz,2H), 4.43 (s, 6H), 3.23 (m, 6H), 2.93 (s, 3H),1.83 (m, 2H), 1.74 (m, 4H), 1.59 (m, 2H), 1.51 (m, 2H), 1.30 (m, 2H), 1.24 (m, 2H). ^{13}C NMR (500 MHz, DMSO-d6): 153.30, 146.30, 144.65, 144.38, 142.68, 128.37, 127.42, 124.86, 124.82, 123.25, 122.30, 115.1, 111.1, 60.40, 46.97, 28.89, 26.45, 26.19, 21.39, 21.13, 19.73.
EA ($C_{40}H_{49}I_3N_4$•H_2O): calc. (obs %) C: 49.71 (49.69), H: 5.11 (5.08), N: 5.80 (5.74).

3,6-Dibromo-9-(1-bromooctyl) carbazole (2-8C-P) (Yield: 72%, Mw: 516.11), ^{1}H NMR (400 MHz, $CDCl_3$) δ: 8.08 (d, *J* = 2.0Hz, 2H), 7.50 (dd, *J* = 2.0, 8.8 Hz, 2H), 7.23 (d, *J* = 8.4Hz, 2H), 4.21 (t, *J* = 7.2Hz, 2H), 3.35 (t, *J* = 7.2Hz, 2H), 1.81 (m, 4H), 1.34 (m, 2H), 1.27 (m, 2H), 1.22 (m, 4H).

3,6-Dibromo-9-(1-(piperidin-1-yl)octyl) carbazole (3-8C-P) (Yield: 76%, Mw: 520.34), ^{1}H NMR (400 MHz, $CDCl_3$) δ: 8.12 (d, *J* = 2.0Hz, 2H), 7.53 (dd, *J* = 2.0, 8.4Hz, 2H), 7.25 (d, *J* = 8.4Hz, 2H), 4.21 (t, *J* = 7.6Hz, 2H), 2.34 (m, 4H), 2.25 (t, *J* = 8.4Hz, 2H), 1.80 (t, *J* = 8.4Hz, 2H), 1.62 (m, 4H), 1.51 (m, 4H), 1.42 (m, 4H), 1.27 (m, 4H).

3,6-Bis-(2-vinylpyridine)-9-(1-(piperidin-1-yl)octyl) carbazole (4-8C-P) (Yield: 68%, Mw: 568.79), ^{1}H NMR (400 MHz, $CDCl_3$) δ: 8.63 (d, *J* =4.8 Hz, 2H), 8.32 (d, *J* =2 Hz, 2H),7.84 (d, *J* =16 Hz, 2H), 7.75 (dd, *J* = 2.0, 8.8 Hz, 2H), 7.67 (dd, *J* = 2.0, 7.6 Hz, 2H), 7.44 (d, *J* = 8 Hz, 2H), 7.38 (d, *J* = 8.8 Hz, 2H), 7.24 (d, *J* = 16 Hz, 2H), 7.14 (dd, *J* = 4.4, 7.2 Hz, 2H), 4.28 (t, *J* = 7.2 Hz, 2H), 2.35 (m, 4H), 2.25(t, *J* = 7.6 Hz, 2H), 1.88 (t, *J* = 7.2 Hz, 2H), 1.58 (m, 4H), 1.42 (m, 4H), 1.35 (m, 4H), 1.26 (m, 4H).

3,6-Bis-(1-methyl-2-vinylpyridium iodide)-9-(1-(1-methyl-piperidinium iodide)octyl) carbazole (*o*-BMVC-8C-P) (Yield: 90%, Mw: 994.61), ^{1}H NMR (400 MHz, DMSO-d6) δ: 8.96 (s, 2H), 8.80 (d, *J* = 8.4Hz, 2H), 8.49 (td, *J* = 2.0, 7.6 Hz, 2H), 8.19 (d, *J* = 16Hz, 2H), 8.04 (d, *J* = 8.8Hz, 2H), 7.87 (t, *J* = 6.8Hz, 2H), 7.82 (d, *J* = 8.8Hz, 2H), 7.69 (d, *J* = 16Hz, 2H), 4.52 (t, *J* = 6.8 Hz,2H), 4.45 (s, 6H), 3.24 (m, 6H), 2.95 (s, 3H),1.83 (m, 2H), 1.74 (m, 4H), 1.59 (m, 2H), 1.50 (m, 2H), 1.30 (m, 4H), 1.24 (m, 4H). ^{13}C NMR (500 MHz, DMSO-d6): 153.30, 146.29, 144.66, 144.38,

142.69, 128.36, 127.38, 124.85, 124.81, 123.22, 122.31, 115.06, 111.07, 60.40, 47.00, 29.09, 28.93, 26.84, 26.25, 21.35, 21.16, 19.74.

EA ($C_{42}H_{53}I_3N_4$•H_2O): calc. (obs %) C: 49.38 (49.37), H: 5.52 (5.50), N: 5.48 (5.44).

3,6-Dibromo-9-(1-bromononyl) carbazole (2-9C-P) (Yield: 70%, Mw: 530.13), ^1H NMR (400 MHz, $CDCl_3$) δ: 8.13 (d, *J* = 2.0Hz, 2H), 7.54 (dd, *J* = 2.0, 8.8 Hz, 2H), 7.26 (d, *J* = 8.4Hz, 2H), 4.20 (t, *J* = 7.2Hz, 2H), 3.35 (t, *J* = 7.2Hz, 2H), 1.80 (m, 4H), 1.35 (m, 2H), 1.28 (m, 4H), 1.22 (m, 4H).

3,6-Dibromo-9-(1-(piperidin-1-yl)nonyl) carbazole (3-9C-P) (Yield: 75%, Mw: 534.37), ^1H NMR (400 MHz, $CDCl_3$) δ: 8.13 (d, *J* = 2.0Hz, 2H), 7.54 (dd, *J* = 2.0, 8.8 Hz, 2H), 7.26 (d, *J* = 8.8Hz, 2H), 4.22 (t, *J* = 7.2Hz, 2H), 2.35 (m, 4H), 2.23 (t, *J* = 8.0Hz, 2H), 1.81 (t, *J* = 8.0Hz, 2H), 1.57 (m, 4H), 1.43 (m, 4H), 1.29 (m, 4H), 1.23 (m, 6H).

3,6-Bis-(2-vinylpyridine)-9-(1-(piperidin-1-yl)nonyl) carbazole (4-9C-P) (Yield: 64%, Mw: 582.82), ^1H NMR (400 MHz, $CDCl_3$) δ: 8.62 (d, *J* = 4.8 Hz, 2H), 8.31 (s, *J* = 2 Hz, 2H), 7.83 (d, *J* = 16 Hz, 2H), 7.74 (dd, *J* = 2.0, 8.8 Hz, 2H), 7.67 (td, *J* = 2.0, 7.6 Hz, 2H), 7.44 (d, *J* = 8 Hz, 2H), 7.38 (d, *J* = 8.4 Hz, 2H), 7.23 (d, *J* = 16 Hz, 2H), 7.13 (dd, *J* = 4.4, 7.2 Hz, 2H), 4.28 (t, *J* = 7.2Hz, 2H), 2.35 (m, 4H), 2.23 (t, *J* = 8.0Hz, 2H), 1.81 (t, *J* = 8.0Hz, 2H), 1.57 (m, 4H), 1.43 (m, 4H), 1.29 (m, 4H), 1.23 (m, 6H).

3,6-Bis-(1-methyl-2-vinylpyridium iodide)-9-(1-(1-methyl-piperidinium iodide)nonyl) carbazole (*o*-BMVC-9C-P) (Yield: 92%, Mw: 1008.64), ^1H NMR (400 MHz, DMSO-d6) δ: 8.89 (d, *J* = 6.4Hz, 2H), 8.83 (s, 2H), 8.58 (d, *J* = 8.4Hz, 2H), 8.49 (td, *J* = 2.0, 7.6 Hz, 2H), 8.18 (d, *J* = 16Hz, 2H), 8.05 (d, *J* = 8.8Hz, 2H), 7.87 (t, *J* = 6.8Hz, 2H), 7.82 (d, *J* = 8.8Hz, 2H), 7.67 (d, *J* = 16Hz, 2H), 4.52 (t, *J* = 6.8 Hz, 2H), 4.43 (s, 6H), 3.26 (m, 6H), 2.95 (s, 3H),1.83 (m, 2H), 1.75 (m, 4H), 1.59 (m, 2H), 1.50 (m, 2H), 1.30 (m, 4H), 1.25 (m, 6H). ^{13}C NMR (500 MHz, DMSO-d6): 153.30, 146.33, 144.65, 144.42, 142.67, 128.05, 127.39, 124.85, 123.15, 122.35, 115.08, 111.12, 60.41, 46.78, 29.29, 29.27, 29.15, 29.00, 26.95, 26.28, 21.40, 21.16, 19.74.

EA ($C_{43}H_{57}I_3N_4$•H_2O): calc. (obs %) C: 50.31 (50.26), H: 5.60 (5.64), N: 5.46 (5.41).

3,6-Dibromo-9-(1-bromododecyl) carbazole (2-12C-P) (Yield: 69 %, Mw: 572.21), ^1H NMR (400 MHz, $CDCl_3$) δ: 8.14 (d, *J* = 2.0Hz, 2H), 7.55 (dd, *J* = 2.0, 8.8 Hz, 2H), 7.27 (d, *J* = 8.4Hz, 2H), 4.24 (t, *J* = 7.2Hz, 2H), 3.39 (t, *J* = 6.8Hz, 2H), 1.82 (m, 4H),

1.39 (m, 2H), 1.25 (m, 14H).

3,6-Dibromo-9-(1-(piperidin-1-yl)dodecyl) carbazole (3-12C-P) (Yield: 68%, Mw: 576.45), ^{1}H NMR (400MHz, $CDCl_3$) δ: 8.14 (d, *J* = 2.0Hz, 2H), 7.55 (dd, *J* = 2.0, 8.4 Hz, 2H), 7.26 (d, *J* = 8.8Hz, 2H), 4.24 (t, *J* = 8.0Hz, 2H), 2.36 (m, 4H), 2.26 (t, *J* = 8.4Hz, 2H), 1.80 (t, *J* = 8.0Hz, 2H), 1.73 (m, 4H), 1.61 (m, 4H), 1.45 (m, 4H), 1.24 (m, 12H).

3,6-Bis-(2-vinylpyridine)-9-(1-(piperidin-1-yl)dodecyl) carbazole (4-12C-P) (Yield: 62%, Mw: 624.9), ^{1}H NMR (400 MHz, DMSO-d6) δ: 8.62 (d, *J* = 4.8 Hz, 2H), 8.31 (d, *J* = 2 Hz, 2H),7.83 (d, *J* = 16 Hz, 2H), 7.74 (dd, *J* = 2.0, 8.8 Hz, 2H), 7.67 (td, *J* = 2.0, 7.6 Hz, 2H), 7.44 (d, *J* = 8 Hz, 2H), 7.38 (d, *J* = 8.4 Hz, 2H), 7.23 (d, *J* = 16 Hz, 2H), 7.13 (dd, *J* = 4.4, 7.2 Hz, 2H), 4.28 (t, *J* = 8.0Hz, 2H), 2.36 (m, 4H), 2.26 (t, *J* = 8.4Hz, 2H), 1.80 (t, *J* = 8.0Hz, 2H), 1.73 (m, 4H), 1.61 (m, 4H), 1.45 (m, 4H), 1.24 (m, 12H).

3,6-Bis-(1-methyl-2-vinylpyridium iodide)-9-(1-(1-methyl-Piperidinium iodide)dodecyl) carbazole (*o*-BMVC-12C-P) (Yield:92%, Mw: 1050.72), ^{1}H NMR (400 MHz, DMSO-d6) δ: 8.86 (d, *J* = 6.4Hz, 2H), 8.81 (s, 2H), 8.58 (d, *J* = 8.4Hz, 2H), 8.49 (td, *J* = 2.0, 7.6 Hz, 2H), 8.19 (d, *J* = 16Hz, 2H), 8.05 (d, *J* = 8.8Hz, 2H), 7.87 (t, *J* = 6.8Hz, 2H), 7.82 (d, *J* = 8.8Hz, 2H), 7.67 (d, *J* = 16Hz, 2H),4.52 (t, *J* = 6.8 Hz, 2H), 4.44 (s, 6H), 3.26 (m, 6H), 2.95 (s, 3H), 1.81 (m, 2H), 1.75 (m, 4H), 1.61 (m, 2H), 1.51 (m, 2H), 1.28 (m, 4H), 1.20 (m, 12H). ^{13}C NMR (500 MHz, DMSO-d6): 153.30, 146.33, 144.65, 144.42, 142.66, 127.93, 127.39, 124.84, 123.12, 122.37, 115.07, 111.14, 60.42, 46.70, 29.53, 29.49, 29.41, 29.33, 29.14, 29.03, 26.35, 21.42, 21.18, 19.73.

EA ($C_{46}H_{65}I_3N_4 \cdot H_2O$): calc. (obs %) C: 50.84 (50.80), H: 6.03 (6.0), N: 5.16 (5.13).

These compounds were dissolved in DMSO for stock solution 1 mM and stored at 4 ℃ and light protection before use. These compounds are stable for several months.

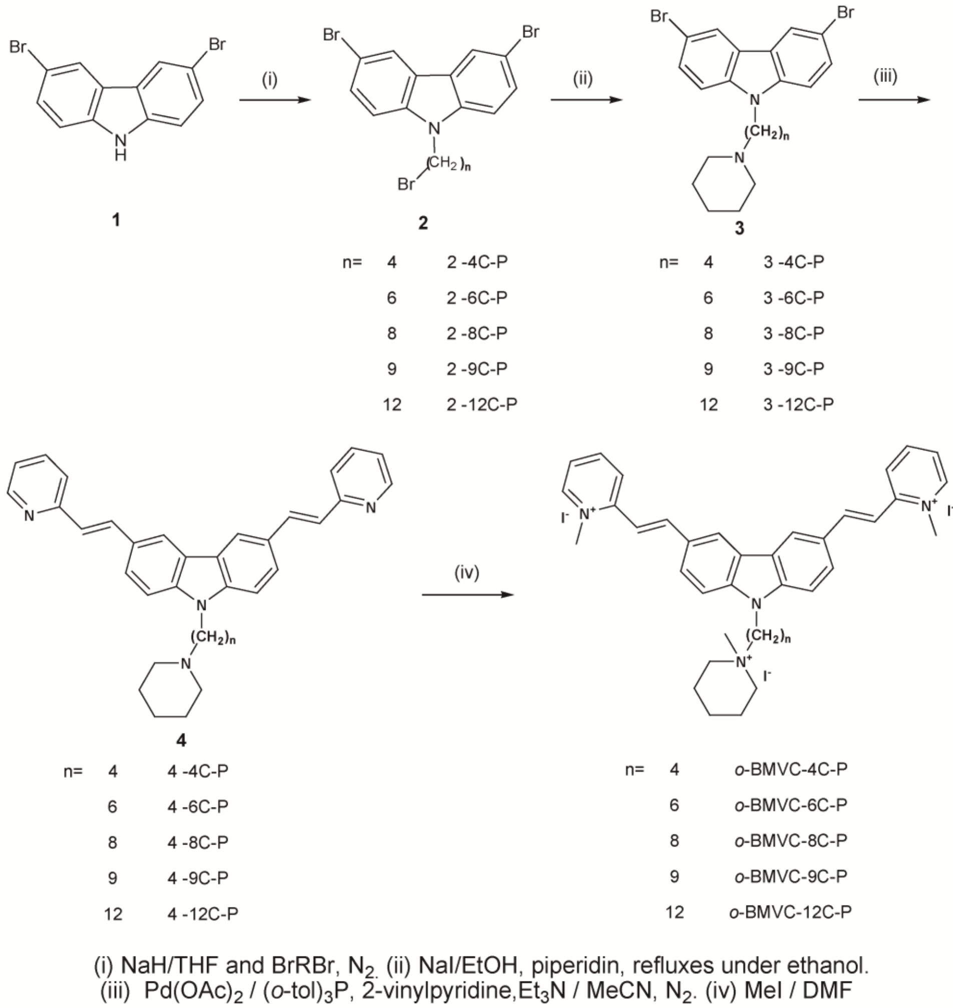

Scheme S1: The syntheses of 9-substituted *o*-BMVC derivatives

Cell lines

CL1-0, human lung carcinoma cell line was kindly provided by Prof. P.-C. Yang (National Taiwan University). Human normal lung fibroblast MRC-5, human normal foreskin fibroblast BJ1, Human normal lung fibroblast IMR-90, human cervical adenocarcinoma HeLa, human lung carcinoma H1299 and human malignant melanoma A375 were obtained from American Type Culture Collection (ATCC). CL1-0 and H1299 were grown in RPMI1640 medium supplied with 10 % fetal bovine serum (FBS) and 1 % antibiotics. A375 was grown in DMEM medium supplied with

10 % FBS and 1 % antibiotics. HeLa, MRC-5, BJ1 and IMR-90 were grown in MEM medium supplied with 10 % FBS and 1 % antibiotics. Antibiotics concentration was 100 U/mL penicillin and streptomycin.

Cell viability analysis

Cells were grown in 96-well plates (2×10^3 cells per well) in a 5% CO_2 incubator at 37 °C. To examine the short-term cytotoxic effect, cells were then treated with different concentrations (0.3125 to 20 μM) of BMVC-12C-P for 72 h. The cytotoxicity was determined using 3-(4,5-dimethylthiazol-2-yl)-2,5-diphenyltetrazolium bromide (MTT) and analyzed by automatic microplate reader (Multiskan EX, Thermo electron corporation, Finland) at 595 nm. The error bars were calculated based on three independent experiments.

For colony formation, cells were seeded into 6 cm tissue culture plate with 1×10^3 cells in each plate for 24 h, then each desired compound at different concentrations were added for 5 d. After that, cells were washed twice with PBS and fixed by 4 % formaldehyde for 2 h at room temperature. After fixation, cells were washed twice with PBS and stained by 0.01 % crystal violet (Sigma, USA) in 25 % methanol for 24 h. Cells were washed twice with PBS and counted.

Population doubling

Cells were treated with 0.5 μM BMVC-12C-P or DMSO (drug vehicle control) and grown in 6 cm tissue culture plates at 5×10^5 cells per plate for 3 or 4 d and then trypsinized and counted. Each time, 5×10^5 cells were replaced in the new culture plate with fresh BMVC derivatives compounds or an equivalent volume of DMSO. The experiments were continued until there were less than 5×10^5 cells available for reseeding. The results were obtained from at least three independent experiments.

Flow cytometry

Cells were incubated with 1 μM *o*-BMVC derivatives for 24 h. Cells were collected by centrifugation and were resuspended in 300 μl PBS, adjusted to a density of approximately 1×10^6 cells per ml, and analyzed by flow cytometry (BD, FACSCalibur). The mean fluorescence intensity at 564-606 nm was measured upon excitation at 488 nm.

DNA preparation

All oligonucleotides were purchased from Bio Basic Inc. (Ontario, Canada) and used without further purification. The oligonucleotides were dissolved in 10 mM Tris-HCl (pH 7.5), followed by heat-denaturation at 95 ^{o}C for 10 min and cooled slowly at 1 ^{o}C/min to room temperature. The annealed oligonucleotides were stored at 4 ^{o}C over night before use. The DNA concentrations were determined by ultraviolet-visible absorption nanophotometer (Implen, Germany).

Absorption and fluorescence

Absorption spectra were taken on a ultraviolet-visible spectrophotometer (HELIOS α, Thermo Fisher Scientific, USA), and fluorescence spectra were recorded on a spectrofluorometer (LS-55, PerkinElmer, USA) with a 2 nm band-pass in a 1 cm cell length of quartz cuvette at room temperature.

Circular dichroism

The CD spectra were averaged 10 scans on a J-815 spectropolarimeter (Jasco, Japan), with a 2 nm bandwidth at 50 nm/min scan speed, and a 0.2 nm step resolution. The CD spectra were measured under N_2 over the range of 210-350 nm to ascertain the G4

structures. The melting curves were recorded at a set temperature controlled by a peltier thermal coupler chamber (PFD-425S/15, Jasco, Japan).

NMR spectroscopy

Experiments were performed using Bruker AVIII 800 MHz spectrometer equipped with a cryoprobe at 25 ℃. The one-dimensional imino proton NMR spectra were measured in H_2O/D_2O (90%/10%) using a WATERGATE pulsed sequence for solvent suppression. The DNA samples were prepared at a strand concentration of 100 μM and specific salt condition with an internal reference of DSS (0.1 mM sodium 4,4-dimethyl-4-silapentane-1-sulfonate).

Polyacrylamide gel electrophoresis (PAGE)

The PAGE was conducted using 20% Polyacrylamide gels. Electrophoresis of the gels was carried out at 25 mA for 3 h at 4 ^{o}C. They were then photographed under ultraviolet light at 254 nm using a digital camera.

Cyclosporin A (CsA) test

For this experiment, HeLa cells were seeded in 12-well plated with coverslip incubated with 1 μM Cyclosporin A (CsA, Sigma, USA) for 24 h to block the mitochondria inner membrane permeability transition pore. After discarded medium, 5 μM BMVC-12C-P was added to incubate with cells for 4 h. Finally, cells were stained with Mitotracker Red and observed by Leica TC5 SP8 confocal microscopy.

Figure S1

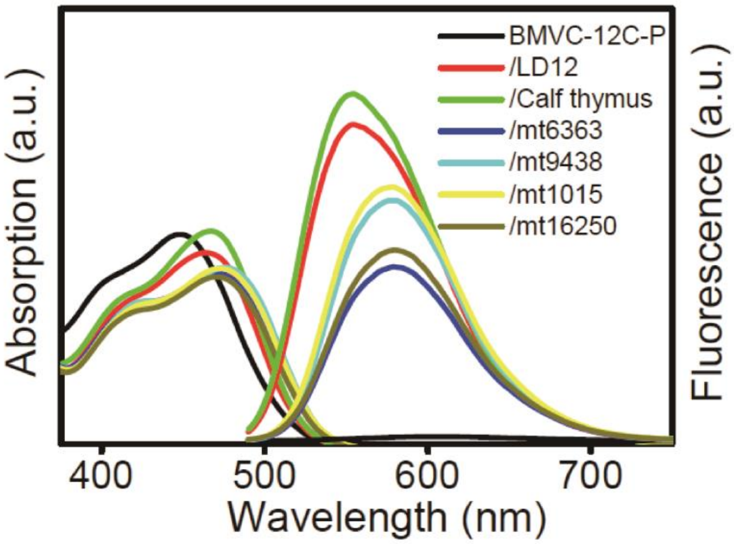

Absorption and fluorescence of 12 μM BMVC-12C-P and its complexes with 6 μM each of LD12, calf thymus, mt6363, mt9438, mt8095, mt1015, and mt16250 in 150 mM K^+ solution. The absorption maximum of BMVC-12C-P at ~450 nm is red-shifted to ~465 nm upon interaction with duplex DNA and further red-shifted to ~475 nm in the presence of G4s. Of importance is that the fluorescence of BMVC-12C-P increases ~70 times upon interaction with duplex DNA and 20-50 times in the presence of G4s.

Figure S2

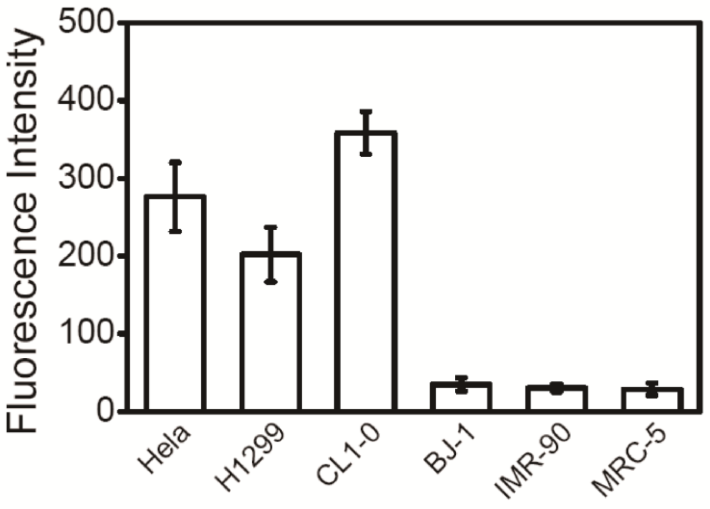

The mean fluorescence intensity of 1 μM BMVC-12C-P incubated with various cancer and normal cell lines for 24 h measured by flow cytometry.

Figure S3

a

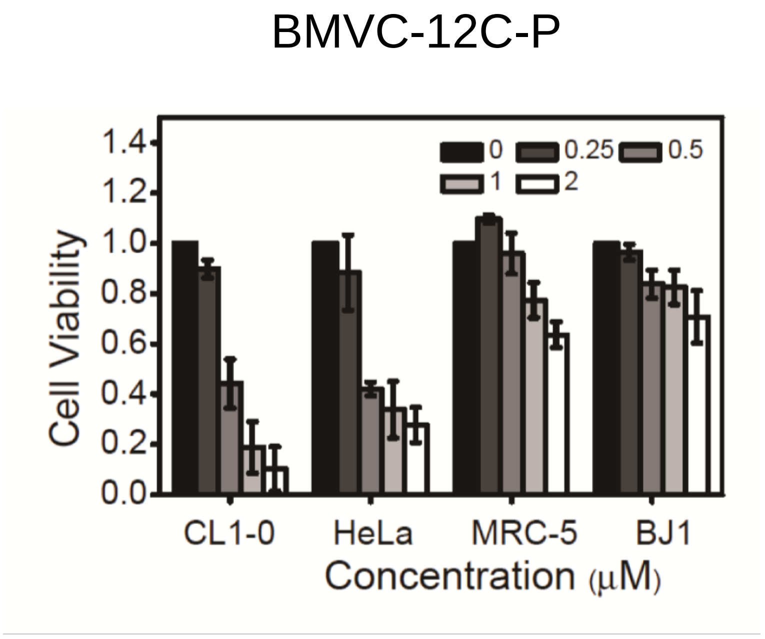

b

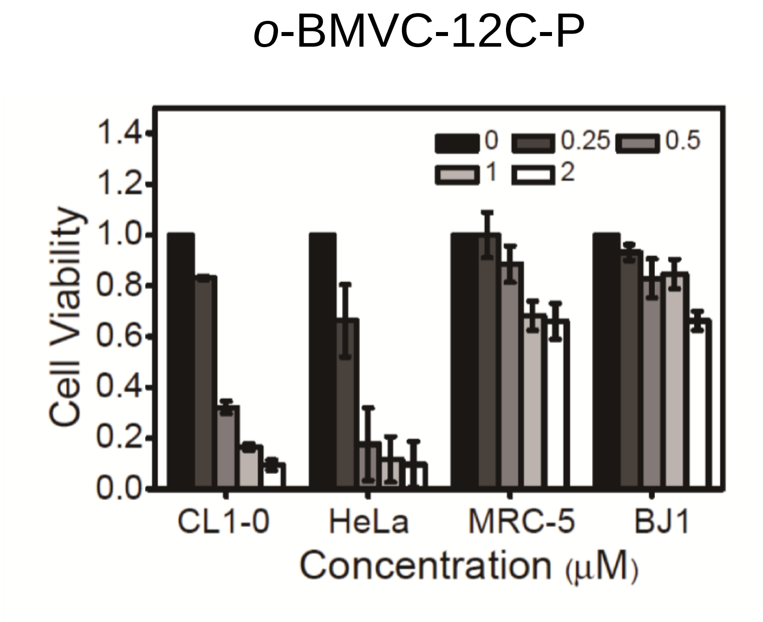

Colony formation assays of **(a)** BMVC-12C-P and **(b)** *o*-BMVC-12C-P on CL1-0 and HeLa cancer cell lines and MRC-5 and BJ1 normal cell lines. Cells (1×10^3) were seeded into each 6 cm culture plate for 24 h, and then added different concentrations of these molecules to corresponding plate for additional 5 days. The data show that *o*-BMVC-12C-P is slightly better anticancer agent than BMVC-12C-P.

Figure S4

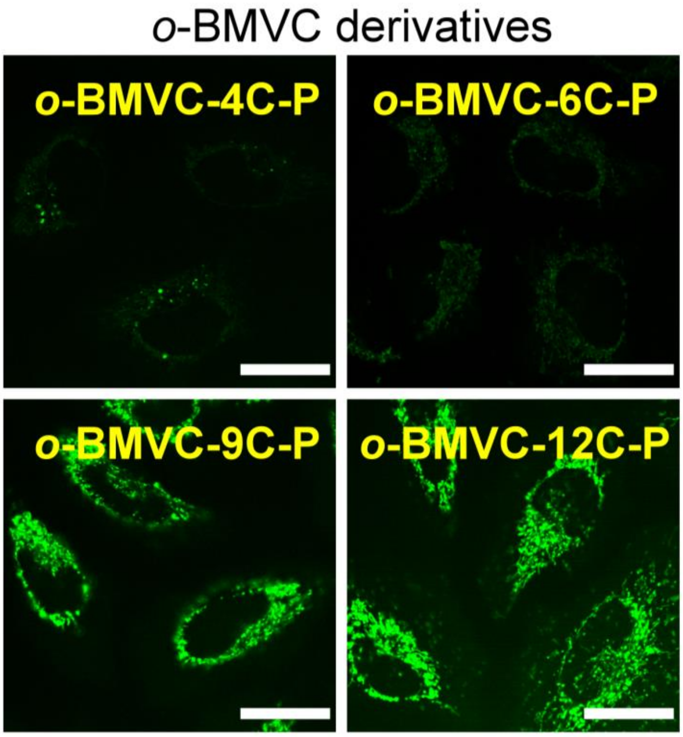

Confocal images of HeLa cells incubated with 5 μM *o*-BMVC derivatives for 24 h. The images were taken under the same condition. Scale bar is 25 μm.

Figure S5

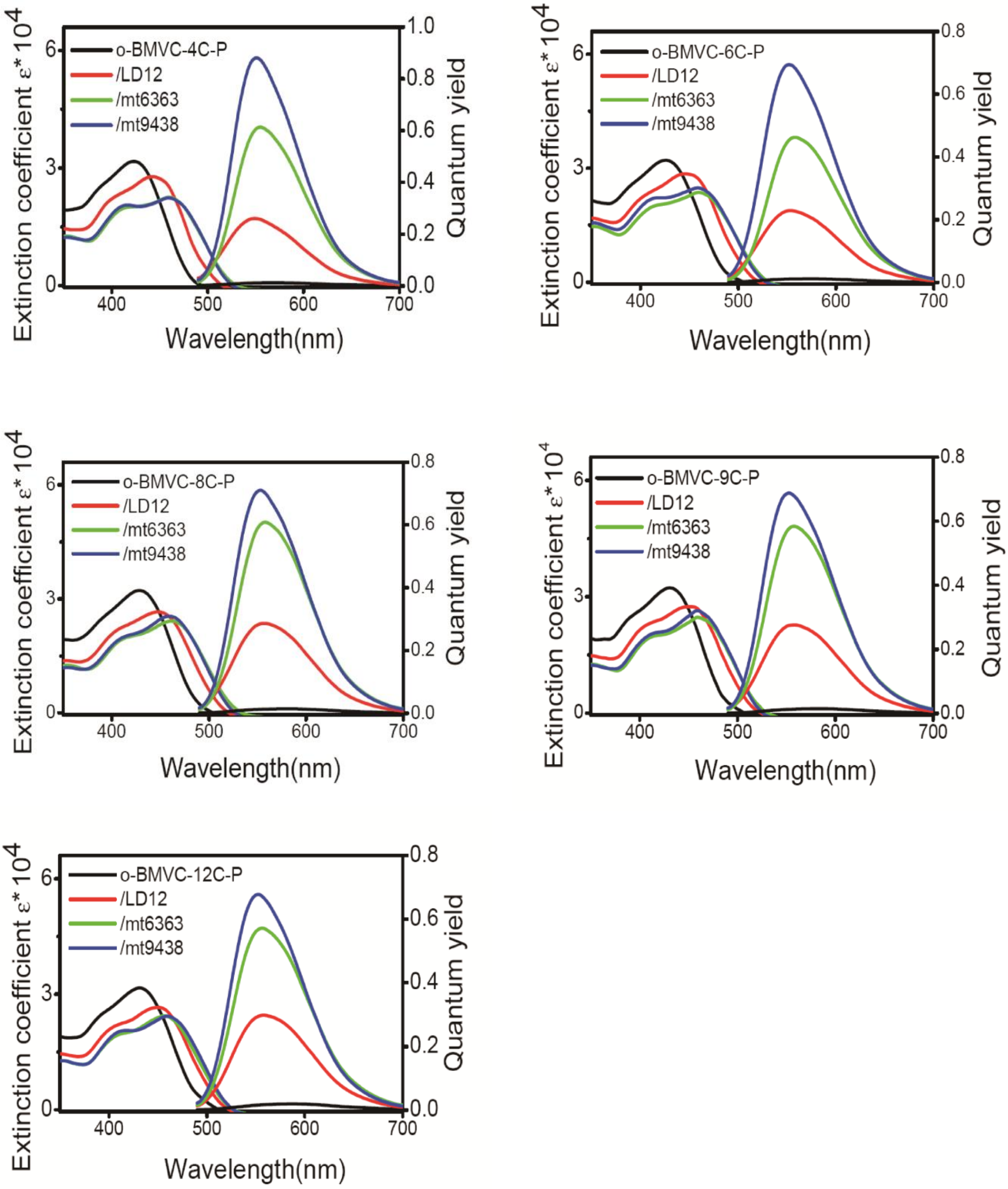

Absorption extinction coefficient and fluorescence quantum yield of each *o*-BMVC derivative and its interaction with LD12, mt6363, and mt9438 DNA.

Figure S6

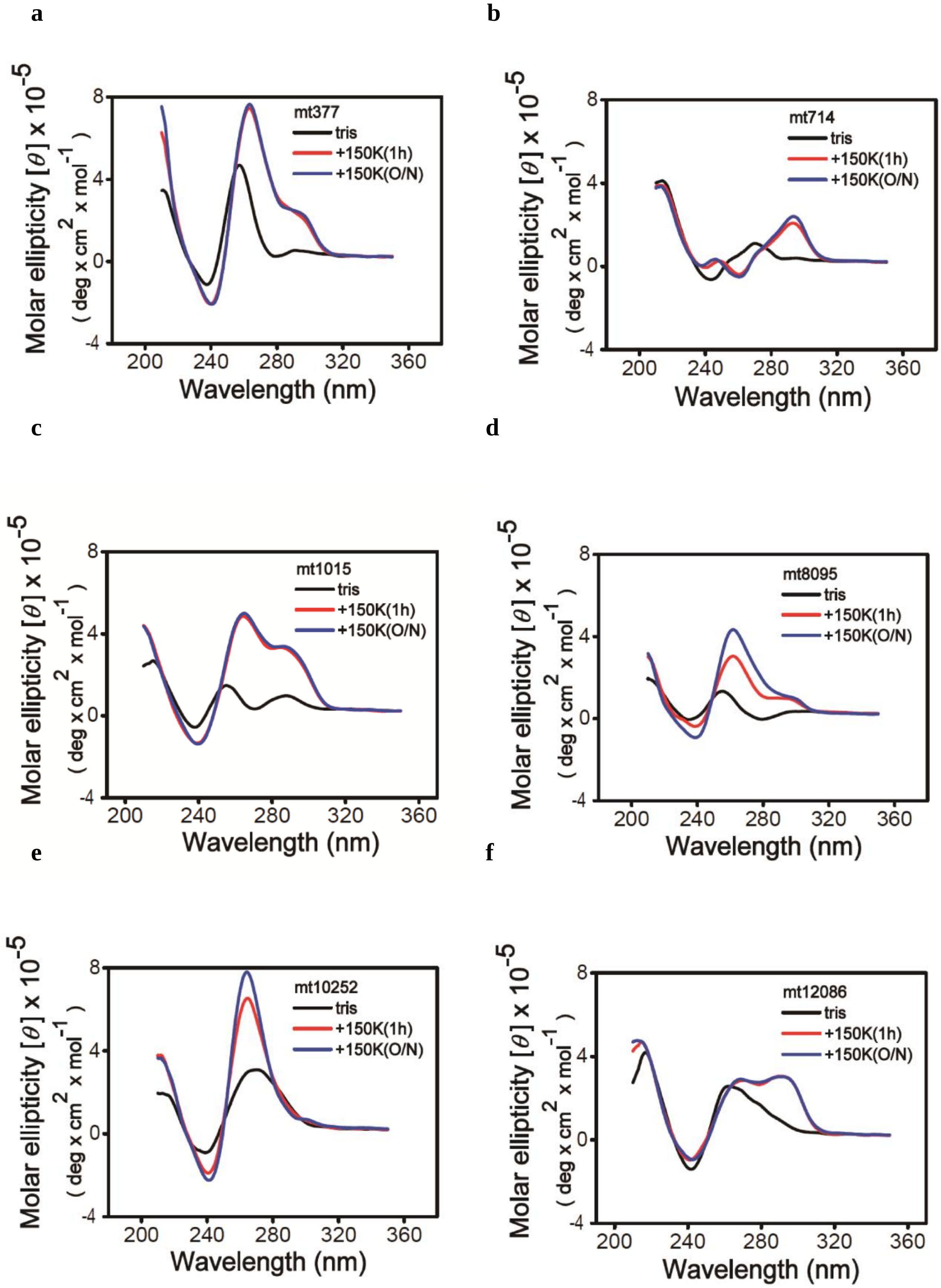
a
b
c
d
e
f
mt377
mt714
mt1015
mt8095
mt10252
mt12086
tris
+150K(1h)
+150K(O/N)
Molar ellipticity [θ] x 10^-5
(deg x cm^2 x mol^-1)
Wavelength (nm)
-4
0
4
8
200
240
280
320
360

g

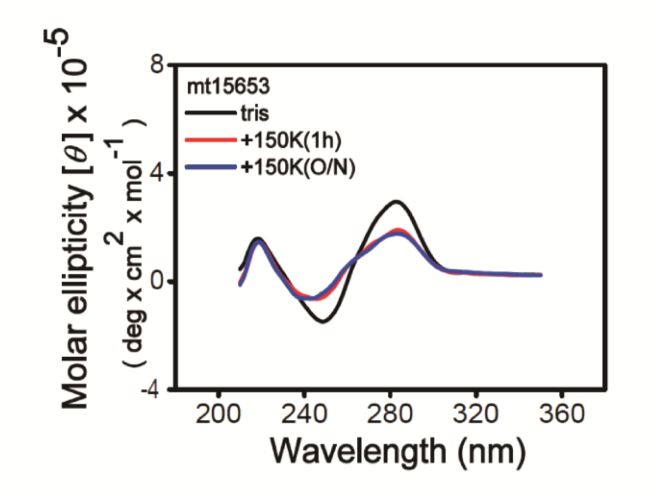

h

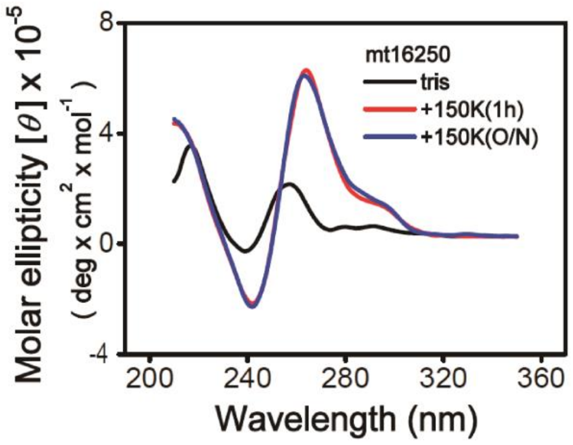

The CD spectra of 4 μM mt377, mt714, mt1015, mt8095, mt10252, mt12086, mt15653, mt16250 **(a-h)** annealed in 10 mM Tris buffer, after the addition of 150 mM K^+ for 1 h, and overnight.

Figure S7

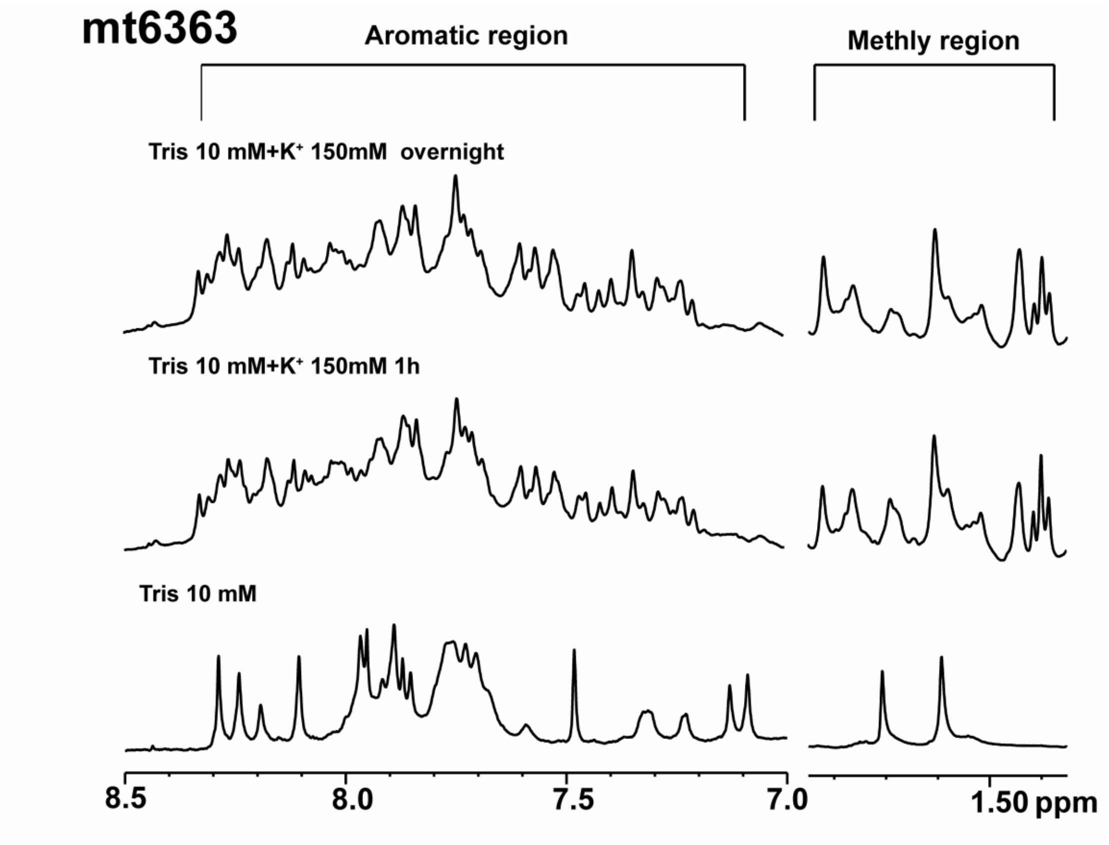

Aromatic and methyl NMR spectra of 100 μM mt6363 annealed in 10 mM Tris buffer, after the addition of 150 mM K^+ for 1 h, and overnight.

Figure S8

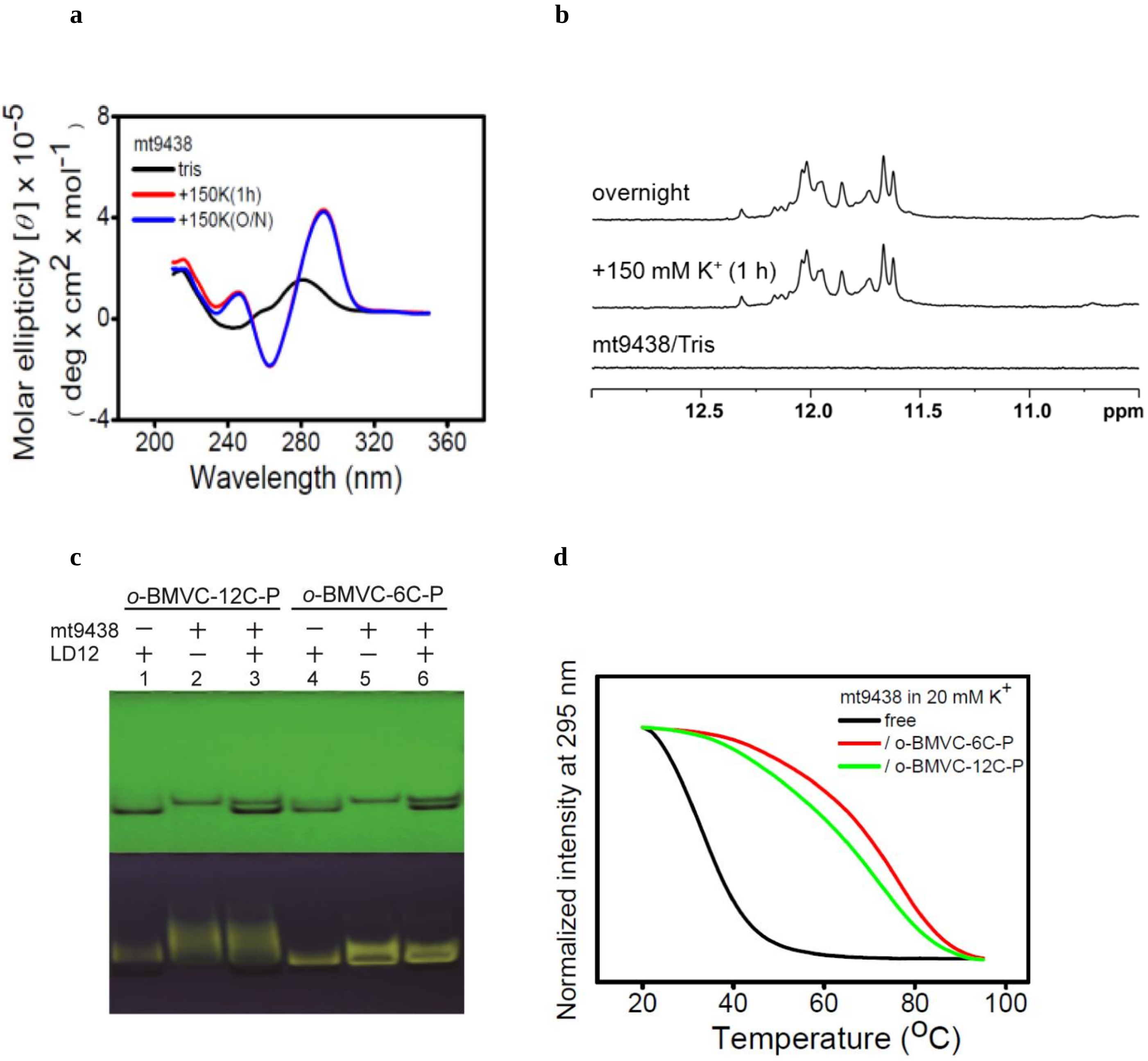

(a) The CD spectra of 4 μM mt9438 and **(b)** NMR spectra of 100 μM mt9438 annealed in 10 mM Tris buffer, after the addition of 150 mM K^+ for 1 h, and overnight. **(c)** UV shadowing of gel assay of (1) LD12 + *o*-BMVC-12C-P, (2) mt9438 + *o*-BMVC-12C-P, (3) LD12 + mt9438 + *o*-BMVC-12C-P, (4) LD12 + *o*-BMVC-6C-P, (5) mt9438 + *o*-BMVC-6C-P, (6) LD12 + mt9438 + *o*-BMVC-6C-P (750 pmol per well). **(d)** The melting curves monitored at 265 nm of 4 μM mt9438 and their complexes with 8 μM *o*-BMVC-6C-P and *o*-BMVC-12C-P in 20 mM K^+ solution.

Figure S9

a

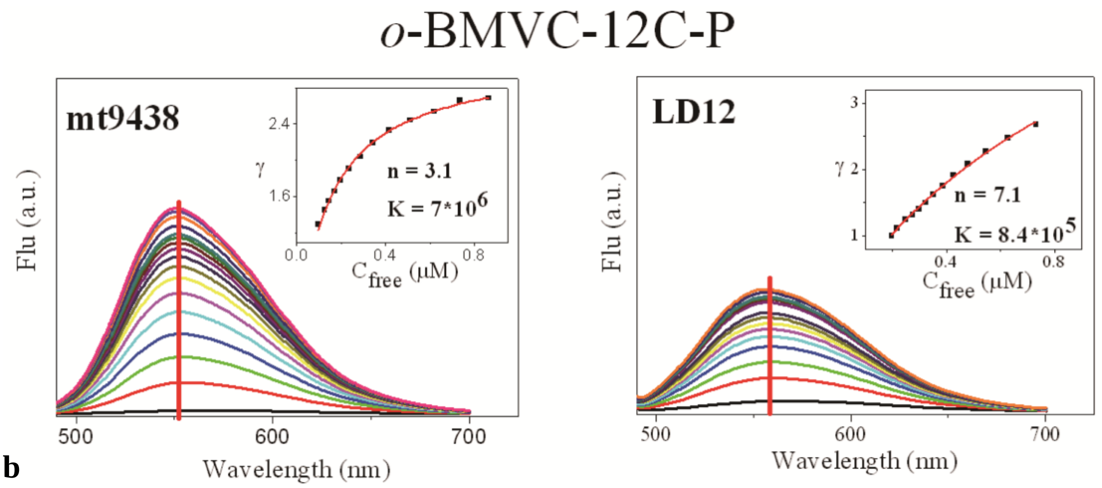

b

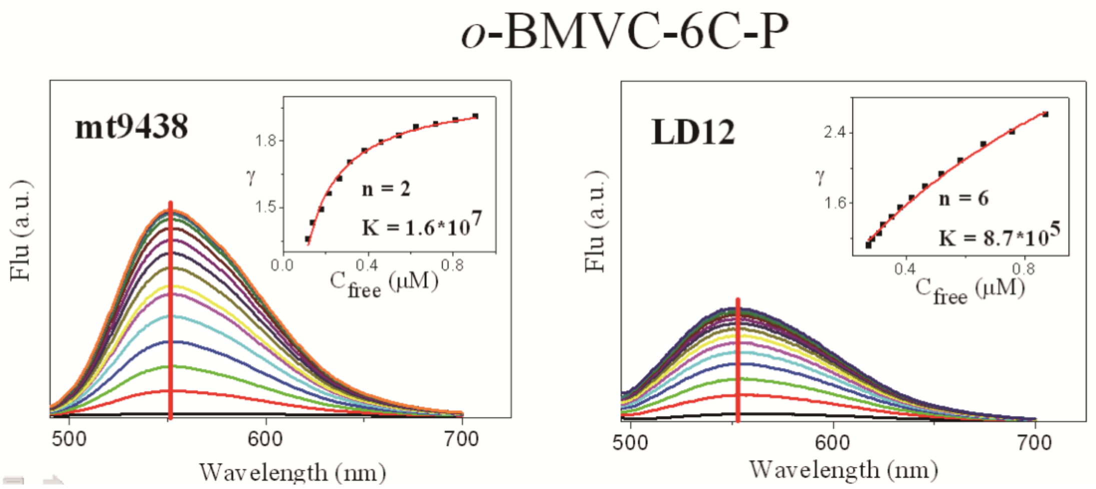

Fluorescence titration of 1 μM *o*-BMVC-12C-P (a) and *o*-BMVC-6C-P (b) by adding mt9438 G4 and LD12 duplex DNA from 0.05 to 0.8 μM in 150 mM K^+ solution. The inset shows the binding plots of γ versus C_f for the titration based on the multiple-equivalent-site model [Methods Enzymol. 340, 3 (2001)]. The binding ratio γ is defined as C_b/C_{DNA}, where C_f, C_b, and C_{DNA} are the molar concentrations of free ligand, bound ligand, and DNA, respectively. The difference between C_t and C_b gives the magnitude of C_f, where C_t is the total concentration of ligand. Using $\gamma = nKC_f/(1+KC_f)$, one can obtain the equilibrium binding parameter K and the average number of ligands bound per each DNA structure.

Figure S10

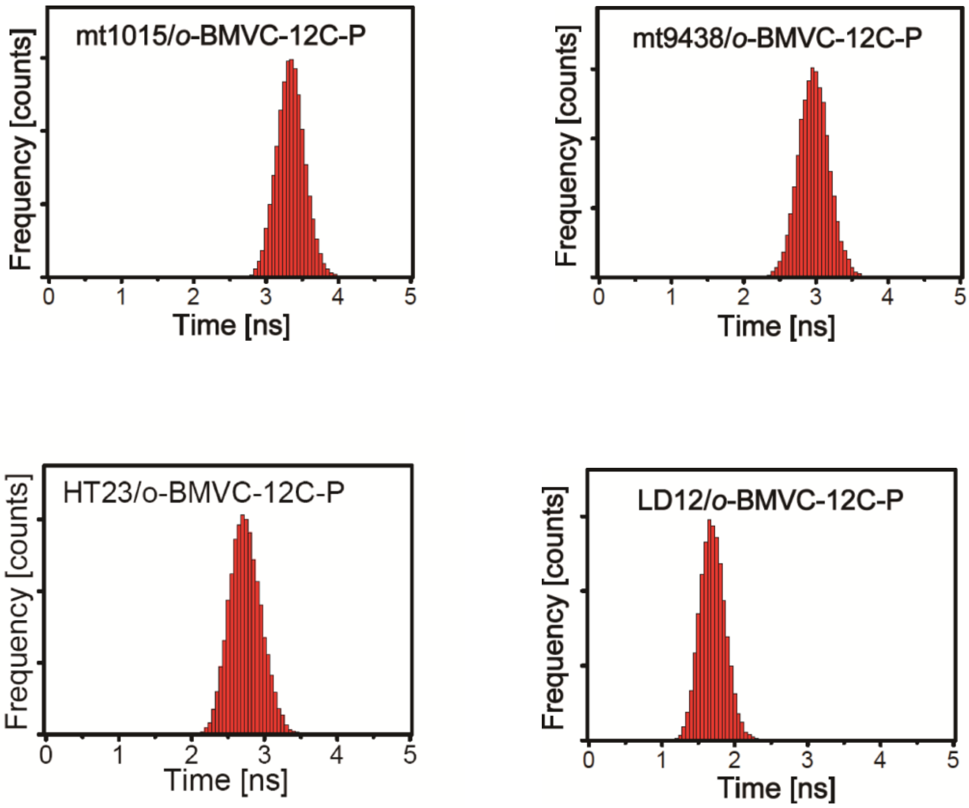

The histograms of the fluorescence decay time of 0.2 μM *o*-BMVC-12C-P upon interaction with mt1015 G4, mt9438 G4, HT23 G4, and LD12 duplex DNA.

Figure S11

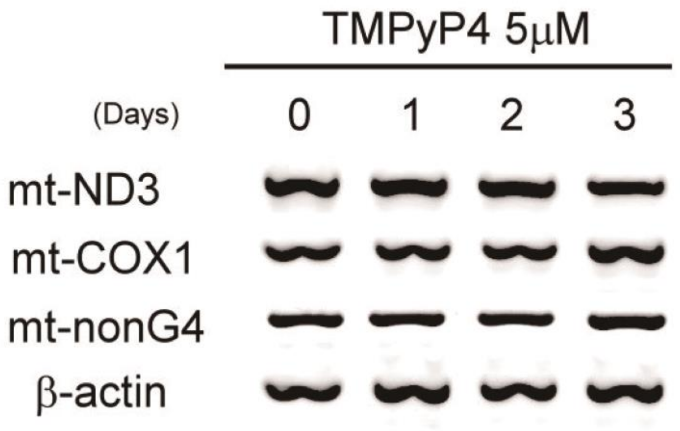

RT-PCR was used to evaluate the suppression of ND3 and COX I expression by TMPyP4. HeLa cells were incubated with TMPyP4 for 3 d. Here β-actin and a non-G4 forming gene sequence were used as controls.

Table S1

Primers and sequences of mitochondria DNA studied in RT-PCR and PCR stop assay.

Sequence (5’→3’)	Abbreviations	Location
5’-AAAATCCACCCCTTACGAGT	mtND3-F	10139-10619
5’-TATTGGCTAAGAGGGAGTGG	mtND3-R	
5’-GCCTGACTGGCATTGTATTA	mtCOXI-F	6899-7439
5’-GGTTCGATTCCTTCCTTTTT	mtCOXI-R	
5’-ATCATAGCAGGCAGTTGAGG	mtND2-F	4919-5399
5’-GGGTGGGTTTTGTATGTTCA	mtND2-R	
5’-TAGGTCTCCACCCCTGACTC	mtND5-F	13019-13559
5’-TAGATAGGGCTCAGGCGTTT	mtND5-R	
5’-CCCGAGCAATCTCAATTACA	mtND6-F	14159-14639
5’-CCGTGCGAGAATAATGATGT	mtND6R	
5’-CCTGTATGCCCTTTTCCTAA	mtCOXII-F	7679-8159
5’-TTTGCTCCACAGATTTCAGA	mtCOXII-R	
5’-TACTACCGTATGGCCCACCA	mtATP8-F	8379-8560
5’-GGGGCAATGAATGAAGCGAA	mtATP8-R	
5’-CCCATTCCGATAAAATCACC	mtCYT B-F	15383-15876
5’-CTACAAGGACAGGCCCATTT	mtCYT B-R	
5’-TCGAGTCTCCCTTCACCATT	mt nonG4-F	9599-10179
5’-GCACTCGTAAGGGGTGGAT	mt nonG4-R	
5’-GTGGGGCGCCCCAGGCACCA	β-actin-F	Internal control
5’-GTCCTTAATGTCACGCACGATTTC	β-actin-R	
5’-GGCGTAGGTTTGGTCTAGGG	mt9438	7113-7132
5’-GGCGTAGATTTGATCTAGGG	mt9438-mut	
5’-TTCTCAGGCTACACCCTAGA	mt9438-R	
5’-AGGGACGCGGGCGGGGGATATAGGGT	mt6363	10182-10207
5’-AGGGACGCAAGCGAATAATATAGGGT	mt6363-mut	
5’-CGAGTGCGGCTTCGACCCTAT	mt6363-R	
